# *Rhinocladiella similis*: A Model Eukaryotic Organism for Astrobiological Studies on Microbial Interactions with Martian Soil Analogs

**DOI:** 10.1021/jacsau.4c00869

**Published:** 2024-12-23

**Authors:** Alef dos Santos, Júnia Schultz, Isabella Dal’Rio, Fluvio Molodon, Marilia Almeida Trapp, Bernardo Guerra Tenório, Jason E. Stajich, Marcus de Melo Teixeira, Eduardo Jorge Pilau, Alexandre Soares Rosado, Edson Rodrigues-Filho

**Affiliations:** † Department of Chemistry, Federal University of São Carlos, São Carlos 13565-905, Brazil; ‡ Biological and Environmental Science and Engineering Division (BESE), King Abdullah University of Science and Technology (KAUST), Thuwal 23955, Saudi Arabia; § Paulo de Góes Microbiology Institute, Federal University of Rio de Janeiro, Rio de Janeiro 21941-902, Brazil; ∥ Oceanographic Institute, University of São Paulo, São Paulo 05508-120, Brazil; ⊥ Analytical Core Lab, King Abdullah University of Science and Technology (KAUST), Thuwal 23955, Saudi Arabia; # Department of Chemistry, State University of Maringá, Maringá 13565-905, Brazil; ¶ Department of Microbiology and Plant Pathology, University of California-Riverside, Riverside 92521, California, United States; ∇ School of Medicine, University of Brasilia, Brasilia 70910-900, Brazil; ○ Bioscience Program, Biological and Environmental Science and Engineering Division (BESE), King Abdullah University of Science and Technology (KAUST), Thuwal 23955, Saudi Arabia

**Keywords:** space exploration, mass spectrometry, black yeast, biosignatures, omics, extremophiles

## Abstract

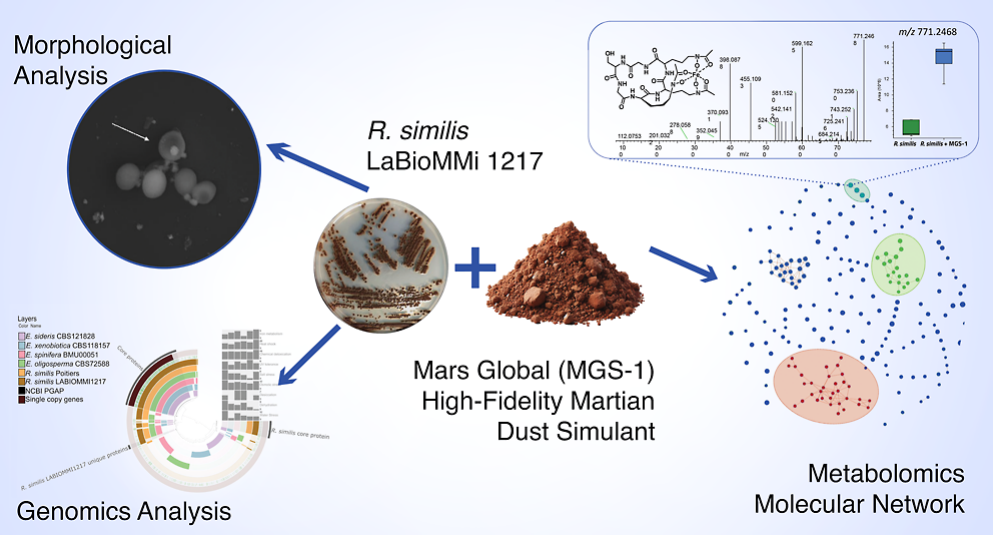

The exploration of our solar system for microbial extraterrestrial life is the primary goal of several space agencies. Mars has attracted substantial attention owing to its Earth-like geological history and potential niches for microbial life. This study evaluated the suitability of the polyextremophilic fungal strain *Rhinocladiella similis* LaBioMMi 1217 as a model eukaryote for astrobiology. Comprehensive genomic analysis, including taxonomic and functional characterization, revealed several *R. similis* genes conferring resistance to Martian-like stressors, such as osmotic pressure and ultraviolet radiation. When cultured in a synthetic Martian regolith (MGS-1), *R. similis* exhibited altered morphology and produced unique metabolites, including oxylipins, indolic acid derivatives, and siderophores, which might be potential biosignatures. Notably, oxylipins were detected using laser desorption ionization mass spectrometry, a technique slated for its use in the upcoming European Space Agency ExoMars mission. Our findings enhance the understanding of extremophilic fungal metabolism under Martian-like conditions, supporting the potential of black yeasts as viable eukaryotic models in astrobiological studies. Further research is necessary to validate these biosignatures and explore the broader applicability of *R. similis* in other extraterrestrial environments.

## 1 Introduction

As space exploration advances, the search for signs of previous or existing life on other celestial bodies has become a major challenge.^1^ In astrobiology, the quest for extraterrestrial life faces significant technical limitations in onsite analysis because of the difficulty of sending human resources. Currently, the most viable approach to determining extraterrestrial life is the detection of biosignatures using analytical techniques (e.g., Raman spectroscopy, Fourier transform infrared spectroscopy, and mass spectrometry (MS)) carried on rovers and landers. These biosignatures range from small molecules, such as O_2_, to complex organic compounds, such as amino acid derivatives, DNA fragments, membrane fatty acids, and secondary metabolites.^2−4^

In our solar system, Mars is the most promising candidate for hosting microbial life. Because the physical and chemical conditions of certain regions on Mars are similar to those on Earth, these locations may potentially harbor extremophilic microbes, making them focal points for future robotic and human exploration.^5^ Compared with the icy moons of the outer solar system, Mars’ proximity to Earth makes it the most accessible destination for space exploration. Consequently, Mars has been the target of important missions, such as the Perseverance rover from the Mars 2020 mission, and the ExoMars mission, which is currently planning to launch the Rosalind Franklin rover in 2028, with the goal of detecting past or present life.^4,6^ Although the surface conditions on Mars are harsh, its subsurface is a potential habitat because it is shielded from ionizing radiation and contains stable deposits of briny liquid water.^4,7^

Earth is the only planet confirmed to sustain life. Thus, investigations of biological molecules produced by terrestrial microorganisms under conditions simulating those on celestial bodies of interest, such as Mars, are crucial.^8^ Extremophiles are of particular interest because they have evolved to thrive under hostile environmental conditions.^9^ On Earth, these microorganisms have been isolated from extreme environments that resemble Martian conditions, including the Atacama Desert, Antarctic Dry Valleys, and Death Valley, and artificial environments like chemically contaminated areas and space station modules.^8,10,11^ Extremophilic bacteria and archaea isolates from these environments have been used as models for understanding potential extraterrestrial life forms.^12,13^ However, because of their complexity and later evolutionary emergence, fungi have only recently been used as models for studying extraterrestrial life forms.^14^

Fungi play crucial roles in extreme environments, particularly in nutrient recycling and soil microorganism modulation.^15^ Characterization of novel extremophilic fungi and analysis of their activities under Martian-like conditions (atmosphere, geochemistry, and temperature) provide insights into potential life on Mars. Black yeasts are of particular interest for studies on eukaryotic survival under Martian conditions because their high melanin pigmentation levels and diverse nature indicate significant astrobiological potential.^16,17^ However, investigations of the genetic and metabolic mechanisms responsible for their adaptability and potential biosignatures under Martian conditions are scarce.

In this context, metabolomics and genomics are powerful tools for understanding the survival mechanisms of black fungi on Mars and detecting biosignatures indicative of life. Thus, an omics approach will expand our knowledge of these microorganisms’ survival strategies and interactions with the Martian environment, thereby enabling the identification of potential biomarkers on other planets,^18^ particularly regarding molecular aspects. Using metabolomics and MS techniques, we can analyze metabolites to identify specific biosignatures that can be targeted for the search for life on Mars.

In this study, we characterized a polyextremophilic black yeast using a multiomics approach to elucidate the molecular mechanisms of survival when cultured in media containing high-fidelity synthetic Martian regolith (MGS-1), thereby simulating microorganism–soil interactions to investigate the resulting biosignatures. Using a combination of genomics, metabolomics, and MS, we identified siderophores, oxylipins, and indole-related compounds as potential biosignatures for astrobiological purposes.

## 2 Material and Methods

### 2.1 Black Yeast Collection, Isolation, and Culture Conditions

Fungal isolation was initially reported by Dos Santos and Rodrigues-Filho.^19^ The black yeast strain analyzed in this study was discovered in an amber flask containing an aqueous HCl solution (pH 1.5) that had been stored in our laboratory (Laboratório de Bioquímica Micromolecular de Microorganismos [LaBioMMi], São Carlos, Brazil). An aliquot (100 μL) of this HCl solution was transferred into Petri dishes containing Czapek Dox medium and incubated at 25 °C for 5 days. Subsequently, viable colonies were isolated on semisolid potato dextrose agar (PDA). Pure colonies were preserved in a 15% glycerol/H_2_O solution and were stored in our local collection under the designated code LaBioMMi 1217.^20^

### 2.2 Morphological Analysis

To phenotypically and morphologically identify the fungus strain LaBioMMi 1217, we inoculated it on two distinct culture media, PDA and Mycosel, to promote growth and incubated the cultures at 25 °C for 15 days. The Mycosel medium was selected to compare it with PDA, in order to observe the dimorphic behavior exhibited by black yeast species. Black yeasts remain in the yeast form when cultured on the Mycosel medium. The colony diameter (mm), structure, pigmentation, and other morphological characteristics were recorded, and colonies were photographed using a smartphone camera.

Next, the colonies were microcultivated.^21^ Briefly, a small block (1 cm × 1 cm) of PDA was placed in the center of a sterile slide. All four sides of the agar were inoculated with the isolate, and a sterile coverslip was gently placed over the agar block. Slide culture was maintained in a moist Petri dish lined with filter paper soaked in sterile water at 25 °C. After 15 days, the fungus had grown onto the coverslip and the slide. The coverslip was then gently removed using sterile forceps and placed on a clean slide for the observation of colony morphology and features using a DMi8 inverted optical microscope (Leica).

### 2.3 Whole Genome Sequencing

We meticulously isolated total genomic DNA from a monosporic colony of black fungus strain LaBioMMi 1217 to conduct a thorough analysis, including precise taxonomic characterization, phylogenetic assessment, diversity profiling, and ecological implications. Briefly, approximately 1 g of wet cells was collected via centrifugation (16,000*g*) and lysed by ultrasound at 70 Hz for 30 min. Subsequently, the genomic DNA was extracted using a Wizard Genomic DNA Purification Kit (Promega, USA). DNA was quantified using a Qubit 4.0 Fluorometer with a Qubit dsDNA HS Assay Kit (Invitrogen, USA), and the quality was assessed by 1% agarose gel electrophoresis stained with SYBR Safe DNA Gel Stain (Invitrogen) and visualized using a iBright Imaging Systems (Thermo Fisher Scientific). The extracted genomic DNA was sent to Macrogen Inc. (Seoul, South Korea) for whole genome sequencing (WGS) using an Illumina platform.

A TruSeq Nano DNA Kit (Illumina) was used to construct paired-end sequencing libraries (2 × 150 bp) with 350 bp insertions. The input material was 110 μg/μL of DNA, according to the manufacturer’s instructions. The quality of the final libraries was assessed using an Agilent 2100 Bioanalyzer (Agilent Technologies). WGS was performed on a NovaSeq 6000 System (Illumina) following the manufacturer’s instructions, achieving a minimum data output of 10 Gb per sample. The raw reads were deposited in the Sequence Read Archive (SRA) repository (accession no. SRR28554191, BioProject PRJNA1005689). The WGS data for the analyzed fungal strain (*Rhinocladiella similis* LaBioMMi 1217) was deposited in GenBank (accession no. JAZDCV000000000, BioProject PRJNA1068593).

The raw reads were filtered using NGS QC Toolkit v2.3^22^ to obtain high-quality (HQ) vectors and adaptor-free reads for genome assembly (HQ cutoff read length, 80%; cutoff quality score, 20). The HQ reads were then used for genome assembly using the Automatic Assembly for the Fungi v0.3.3 pipeline.^23^ First, the Illumina sequencing reads were trimmed using the BBDuk tool of BBMap v38.95 software (https://sourceforge.net/projects/bbmap/). Second, BBMap was used to eliminate contaminant reads. Third, resultant HQ reads were assembled using SPAdes v3.15.4.^24^ Fourth, contaminant contigs were identified and removed using the National Center for Biotechnology (NCBI) tool FCS-GX.^25^ Fifth, duplicated contigs were purged using minimap2,^26^ and the final assemblies were polished using Pilon v1.24 software.^27^ Finally, the scaffolds were sorted by length, and the fasta headers were renamed for further annotation. The assembly quality was verified using QUAST v5.1.0^28^ and completeness by BUSCO using the chaetothyriales_odb10 database.^29^

### 2.4 Multilocus Sequence Analyses

First, we identified the sequence of the *ITS1*–*2* locus of strain LaBioMMi 1217 from the assembled genome using BLASTn analysis and compared it against the BLASTnr database. The initial assessment of the genetic background of the LaBioMMi 1217 strain indicated that it belonged to either *R. similis* or *Exophiala spinifera*. Therefore, we performed multilocus sequence analyses (MLSA) to obtain robust phylogenic data based on the *ITS*, *LSU*, *tef1*, *rpb1*, and β-tubulin loci.^30^ The available sequences of the aforementioned loci belonging to Clade 1 contained members of Herpotrichiellaceae and were retrieved from the NCBI database (Supporting Information Table S4). The *ITS*, *LSU*, *tef1*, *rpb1*, and β-tubulin sequences were identified and retrieved from the LaBioMMi 1217 genome sequence using BLASTn. The individual gene sequences for the five data sets were aligned separately using the PASTA software.^31^

Next, alignment positions were purged using ClipKIT v1.3 with the smart-gap function. The individual alignments were then concatenated for phylogenetic analysis using IQ-TREE v2.1.1 software.^32^ ModelFinder was used to evaluate the best DNA substitution model.^33^ Ultrafast bootstraps and the Shimodaira–Hasegawa-like approximate likelihood ratio test (SH-aLRT) were used for branch support.^34^ Finally, the tree topologies were visualized using the FigTree software (http://tree.bio.ed.ac.uk/software/figtree/).

### 2.5 Genome Annotation and Comparative Genomic Analysis

The assembled LaBioMMi 1217 sequence was annotated using the funannotate v1.8 pipeline.^35^ TANTAN^36^ was used to identify and mask repetitive DNA content using the funannotate mask command.

For gene prediction, we used mRNA molecules isolated from LaBioMMi 1217. Briefly, approximately 500 mg of mycelia cultured on PDA was harvested for total RNA extraction. Mycelial cells were lysed for 30 min using Buffer RLT (Qiagen) and glass beads. Then, we used an RNeasy Kit to extract total RNA according to the manufacturer’s instructions. Approximately 1 μg of total RNA was used as the input material for constructing sequencing libraries using a TruSeq Stranded mRNA Library Prep Kit (Illumina). The quality of the final libraries was assessed using an Agilent 2100 Bioanalyzer. The libraries were sequenced on the NovaSeq 6000 System (2 × 150 bp) according to the manufacturer’s instructions, achieving a minimum data output of 2 Gb per sample. The raw mRNA reads were deposited in the SRA database (accession no. SRR28554245).

Gene models were predicted using evidence-based and ab initio (i.e., structure-based) software. Aiming to find conserved gene models, we used mRNA reads assembled via Trinity and PASA^37^ for training the ab initio predictors Augustus,^29^ GlimmerHMM,^38^ SNAP,^39^ and CodingQuarry.^40^ The self-training GeneMark-ES algorithm was also used with the option for fungal genomes.^41^ The weighted consensus gene structure was obtained via EVidenceModeler^42^ using the following weights: PASA = 6, Augustus HiQ = 2, and CodingQuarry = 2. The remaining predictors were set to 1. Genes <50 amino acids and those identified as transposable elements were excluded from the data set. tRNAscan-SE was used to predict tRNAs.^43^

In addition, the funannotate annotate function was used to annotate genes using eggNOG,^44^ Pfam,^45^ InterPro,^46^ Gene Ontology (GO) terms,^47^ MEROPS,^48^ and the Fungal Secretome Database,^49^ FungiSMASH, the fungal-genome-specific version of antiSMASH 6.0^50^ was used to predict biosynthetic gene clusters.

We also employed a phylogenomic approach using the PHYling v2 pipeline (https://github.com/emmadebayos/PHYling_unified/) with sets of universal single-copy fungal markers available from the BUSCO database.^51^ We then used hidden Markov models to search for each biomarker in the fungi_odb10 database using the hmmalign tool. Subsequently, individual alignments were trimmed using ClipKIT v1.3^52^ with the smart-gap function to remove spurious positions. Next, individual alignments were concatenated to reconstruct the maximum-likelihood species tree using IQ-TREE v2 software.^32^ ModelFinder was used to evaluate each marker’s best protein substitution model.^33^ Branch support was assessed using ultrafast bootstraps^53^ and SH-aLRT.^34^ The tree topology was visualized using FigTree software. Finally, we used the funannotate compare function to perform comparative genomic analyses of *Rhinocladiella* fungi. We counted various genomic features, including Pfam, carbohydrate-active enzymes (CAZymes), MEROPS, transmembrane proteins, secreted proteins, clusters of orthologous groups (COGs), secondary metabolites, and fungal transcription factor domains, plotting each category with a standard deviation >1 in a heat map.

We performed data mining using the functional tables generated by Pfam, InterPro, and NCBI COG. Manual categorization was used to search for genes and metabolites of interest for astrobiology applications, such as genes for ultraviolet (UV) radiation resistance, cold tolerance, osmotic stress tolerance, oligotrophic metabolism, and Fe metabolism (Table S2). To gain a comprehensive understanding of the similarities and differences between the studied strains (our strain and the five closely related strains belonging to the *Rhinocladiella* and *Exophiala* genera) regarding protein prediction with a focus on astrobiological aspects, we generated a pangenome using anvi’o^54^ and the annotated genomes (.gbk files), as previously described. The proteins of each metabolism of interest (Table S2) were counted in a dereplicated manner, plotting the presence and absence of the proteins in each analyzed genome.

### 2.6 LaBioMMi 1217 Culture on Synthetic Martian Regolith Substrate

After culturing LaBioMMi 1217 for 15 days at a controlled temperature of 25 °C on PDA, we used a sterile inoculation loop to transfer a pure colony to a Falcon tube containing 10 mL of 0.9% NaCl. Then, the cell suspension was sequentially diluted to an optical density of 0.7–0.9 at 595 nm (final concentration: 10^7^–10^8^ cells/mL). This cell suspension was used as the inoculum for an experiment to culture LaBioMMi 1217 in a synthetic Martian regolith.^55^ Briefly, 100 μL of the prepared cell suspension was inoculated into 50 mL Erlenmeyer flasks containing 20 mL of potato dextrose broth (PDB; Sigma-Aldrich) and 5 g of synthetic Martian regolith (MGS-1; ratio 1:4 (v/w)) with 1.5% (w/w) magnesium perchlorate.

The experimental control comprised 20 mL PDB inoculated with LaBioMMi 1217. To eliminate molecules originating from the culture medium and MGS-1 during data processing, experimental blanks were used (no inoculum suspension). Subsequently, the flasks were subjected to orbital agitation (150 rpm) and cultured for 10 days at a constant temperature of 25 °C. The experiments were conducted in five replicates.

### 2.7 Scanning Electron Microscopy Analysis

Samples were prepared for scanning electron microscopy (SEM) by transferring 10 μL of each treatment (LaBioMMi 1217 in the presence and absence of MGS-1) to a silicon glass support, followed by fixation in 3% glutaraldehyde for 3 h. The fixed samples were dehydrated in a graded series of isopropanol (35%, 50%, 75%, 90%, and 100%), underwent critical point drying for 5 h, and finally coated with metallic iridium particles. Images were captured using field emission SEM (Merlin Gemini, Zeiss).

### 2.8 Microbial Metabolite Extraction

To investigate the production of secondary fungal metabolites, PDB cultures were transferred from Erlenmeyer flasks to 50 mL Falcon tubes. We added 5 g of MGS-1 to the control experiments. To perform metabolomic analysis without interference from the matrix during extraction, no MGS-1 was added to the blank experiments. The samples were frozen at −80 °C for 24 h and then lyophilized. The metabolites and lipids were extracted using liquid–liquid separation method. Briefly, 20 mL of lysis solution containing MTBE/MeOH (4:1, v/v) was added to each tube, and the cells were lysed by placing the tubes on a vertical shaker for 60 min. Then, 15 mL of H_2_O/MeOH (4:1, v/v) was added, and the tubes were placed in an ultrasonic bath at approximately 20–30 kHz for 20 min. Next, 1 mL of the hydroalcoholic phase from each extract was transferred to an Eppendorf tube and completely dried under a fast vacuum. The dried extracts were reconstituted in 250 μL of acetonitrile (ACN; purity ≥99.9%, Sigma-Aldrich) for liquid chromatography–high-resolution tandem mass spectrometry (LC–HRMS/MS) analysis.

### 2.9 LC–HRMS/MS Analysis

The hydroalcoholic crude extracts underwent untargeted screening and metabolomic analysis using a Vanquish Ultra-High-Performance Liquid Chromatography (UHPLC) system coupled with an ID-X Tribrid mass spectrometer (Thermo Fisher Scientific) equipped with a heated electrospray ionization (ESI) source. Chromatographic separation was performed using an 2.1 × 50 mm × 1.7 μm Acquity UPLC BEH C18 column (Waters Corp.) maintained at 40 °C. The injection volume of each sample was 10 μL. The flow rate was set at 0.5 mL min^–1^, and a 15 min gradient was applied for separation using mobile phases A (H_2_O with 0.1% formic acid) and B (ACN with formic acid 0.1%) under the following conditions: 0–1 min, 5% B; 1–3 min, 5% B to 15% B; and 3–15 min, 15% B to 100% B. Washing was performed using 100% B and equilibrated with 5% A. The MS results were calibrated using an Pierce FlexMix Calibration Solution (Thermo Fisher Scientific) according to the manufacturer’s instructions. Positive-ion mode ESI was applied to the compounds of interest using the following MS parameters: sheath gas flow rate, 40; auxiliary gas flow rate, 20; spray voltage, 4.0 kV; capillary temperature, 350 °C; auxiliary gas heater, 250 °C; S-lens RF level, 50; and resolution, 60,000. We used normalized collision energies of 15, 25, 35, and 45 eV for the MS/MS measurements. Pooled injected samples were used for quality control (QC).

### 2.10 MS Data Processing

The processing workflow was performed using Compound Discoverer (CD) v3.3 software. First, spectra were selected from the raw data, followed by retention time (RT) alignment with a tolerance of 1 min and mass accuracy of 5 ppm. Then, unknown compounds with an RT tolerance of 0.7 min and mass deviation of 5 ppm were detected and grouped. In the second step, a feature table was created and statistical analyses were performed. Confidence with which a compound is named (i.e., annotated) followed the Metabolomics Standards Initiative guidelines,^56^ where the annotation is classified according to four levels: unknown compounds (Level 4, low), putatively characterized compounds (Level 3, medium), putatively annotated compounds (Level 2, high), and identified metabolites (Level 1, highest). Annotation was based on precise mass analysis (error range: 0–2 ppm) and the MS/MS fragmentation profiles from the mzCloud and mzVault databases. Analytical standards were injected to confirm oxylipin detection. The CD workflow included the creation of a molecular network, where the MS/MS spectra of the biomarkers of interest were analyzed and grouped using a coverage of 60, a score of 50, and a fragment match of 25. The images generated by the molecular network were colored manually using InkScape v1.3.2 software.

### 2.11 Effect of MGS-1 on Metabolite Extraction and Experimental Control Design

It is crucial to meticulously design the entire workflow when conducting metabolomics analysis to identify biomarkers. In particular, the extraction process must be considered to prevent biased data interpretation. In the experiment aimed at observing variations in metabolism, the fungus was cultured in the presence of a particulate material (i.e., the synthetic Martian regolith MGS-1). However, the presence of MGS-1 may have facilitated cell lysis during extraction, enhancing the retrieval of metabolites. Therefore, we assessed the influence of MGS-1 on the extraction process to explore its effect on the induction of the molecules. This preliminary assessment guided our selection of the most suitable control experiment for statistical comparison. To this end, crude extracts from all samples were evaluated by ultrahigh-performance (UHPLC–MS/MS), and an unsupervised analysis (principal component analysis, PCA) was performed.

### 2.12 Laser Desorption Ionization Mass Spectrometry Analysis of Microbial Extract-Doped MGS-1

We adapted the method of dos Santos et al.^20^ for the laser desorption ionization mass spectrometry (LDI-MS) analysis of microbial extract-doped MGS-1. Briefly, we added 5 mg of the dried hydroalcoholic extract of the fungus cultured in the previous experiment to 1 mL of LC–MS grade MeOH, transferred the solution to a microtube containing 500 mg of MGS-1, and vortexed at 70 Hz for 5 min. Then, we used a micropipette to transfer 1 μL of each prepared suspension to different points of the sample holder and dried them for 15 min at room temperature.

Measurements were performed using an Autoflex Speed mass spectrometer (Bruker Daltonics, Bremen, Germany). A 355 nm Nd/YAG laser source operating at a frequency of 10 Hz and 70% of the nominal power focused on a point approximately 5 μm in diameter was used to induce ionization/desorption processes. Positive-ion mass spectra were acquired after time-of-flight separation in reflectron mode with an acceleration voltage of 20 kV. All spectra were collected from an average of 100 laser shots. A standard mixture for low masses was used for mass calibration. MTP 384 polished steel target plate and MGS-1 mineral origin matrices were used. The operating pressure of the spectrometer was approximately 10^–7^ mbar.

## 3 Results

### 3.1 Morphological and Molecular Characterization of LaBioMMi 1217

**Figure 1:**
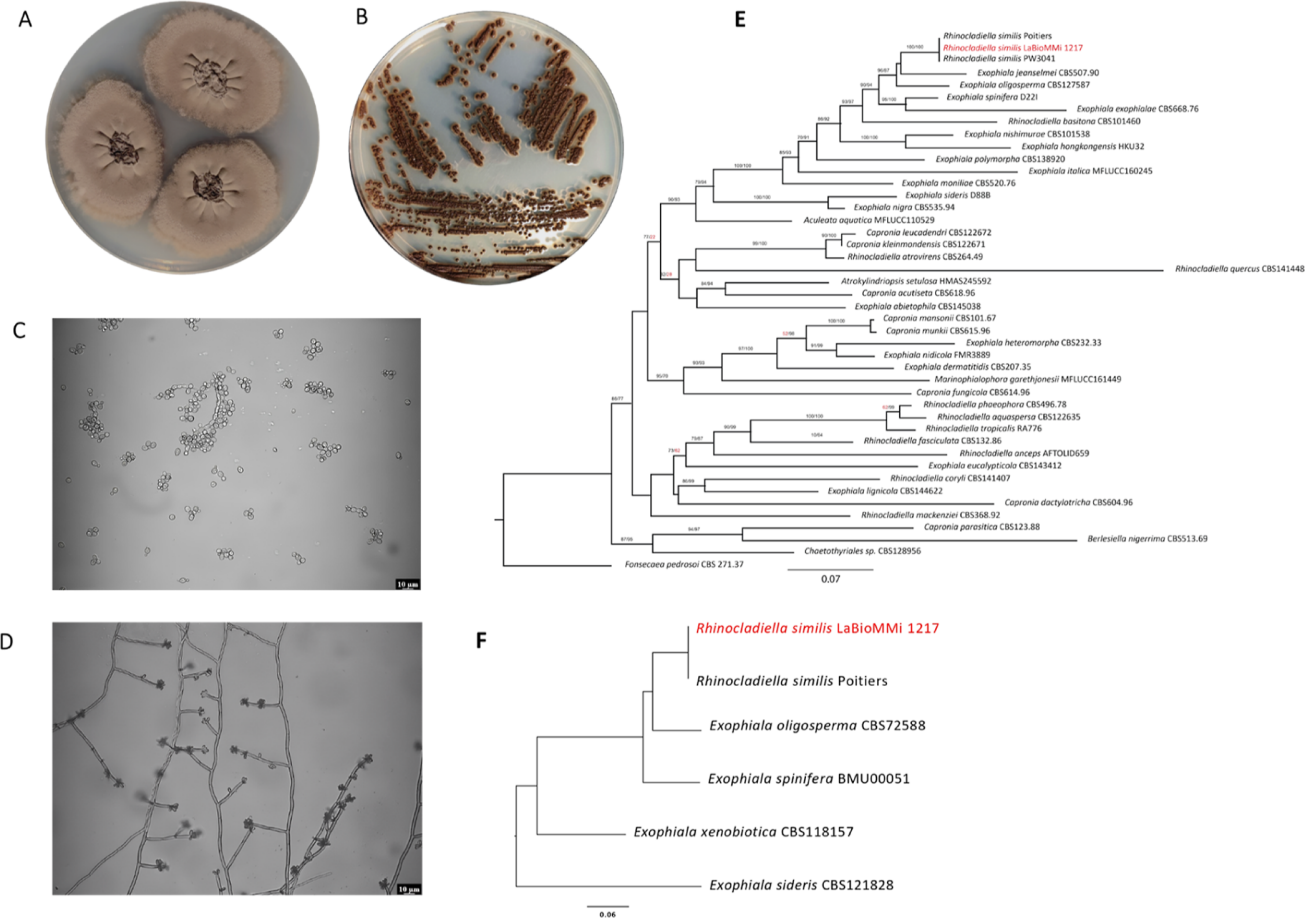
Morphological and molecular characterization of fungal strain LaBioMMi 1217. Macroscopic structure of the fungus cultured on (A) potato dextrose agar for 20 days and (B) Mycosel medium for 15 days. Microscopic structure of the fungus cultured on (C) Mycosel medium for 15 days and (D) potato dextrose agar for 20 days. (E) Concatenated multilocus sequence analysis (MLSA) tree based on five conserved marker genes (*ITS*, *LSU*, *tef1*, *rpb1*, and β-tubulin) extracted from whole genome sequencing (WGS) data from LaBioMMi 1217 or available genes in databases. The MLSA tree was rooted in *Fonsecaea pedrosoi*. (F) WGS-based phylogenomic analysis of *R. similis* using midpoint rooting. We constructed a phylogenomic tree for *Rhinocladiella* and *Exophiala* strains using available genomic sequences.

The morphological characteristics of the fungal strain LaBioMMi 1217 were assessed after 20 and 15 days of culture on PDA (Figure 1A) and Mycosel medium (Figure 1B), respectively. Irregular-shaped brown colonies with a rough texture were observed on PDA, and the periphery had dark greenish-gray pigmentation (Figure 1A). Optical microscopy of the fungus in filamentous form revealed thick-walled, dematiaceous, and septated hyphae containing lateral and apical conidiophores with elongated to ovoid conidia (Figure 1D). In contrast, yeast-like microcolonies, approximately 2 mm in diameter, were observed on Mycosel medium. The colonies had dark brown pigmentation that appeared gelatinous in the center and slightly lighter at the periphery (Figure 1B). Optical microscopy revealed several yeast-like cells, some of which were budding, and pseudohyphae (Figure 1C). These morphological characteristics were consistent with those of the *Rhinocladiella* and *Exophiala* genera.

The molecular typing results for LaBioMMi 1217 indicated that it belonged to *R. similis* based on three criteria. First, the BLAST analysis of the sequenced ITS fragments showed 100% identity with the type strain CBS 111763 (accession no. NR_166008.1). Second, MLSA using five different nuclear markers commonly employed to distinguish species within the order Chaetothyriales revealed that LaBioMMi 1217 formed a monophyletic cluster with other *R. similis* strains and was closely related to other extremotolerant fungi, such as *Exophiala jeanselmei*, *Exophiala oligosperma*, and *E. spinifera* (Figure 1E). Third, phylogenomic analysis (Figure 1F) confirmed the MLSA results, with the *R. similis* strains LaBioMMi 1217 and Poitiers forming a strong dyad that was closely related to the aforementioned *Exophiala* strains.

### 3.2 Genomic Features of *R. similis* LaBioMMi 1217 and Closely Related Strains

The genomic sequence of *R. similis* LaBioMMi 1217 was deposited in GenBank (accession no. JBBMOB000000000, BioProject PRJNA1005689). The *R. similis* LaBioMMi 1217 genome was assembled into 80 scaffolds (total size = 34,715,284 bp), with an average scaffold size of 433,941 bp (N50 = 1,238,920 bp, L50 = 10) and a GC content of 50.98%. Genome annotation revealed 12,908 genes comprising 12,857 protein-coding genes and 51 tRNAs (Table 1).

**Table 1:** Genome Characterization and Assembly Statistics of *Rhinocladiella similis* LaBioMMi 1217 and its Closely Related Strains in the *Exophiala* Genus

feature	R. similis LaBioMMi 1217	R. similis Poitiers	E. xenobiotica CBS118157	E. spinifera BMU00051	E. sideris CBS121828	E. oligosperma CBS72588
total length (bp)	34,715,284	34,259,553	31,405,760	32,380,025	29,505,589	38,224,514
no. of contigs	80	12	15	7	5	143
no. of contigs >100 kb	43	9	7	7	4	22
L50	10	4	3	3	2	5
L90	29	8	7	7	4	17
N50	1,238,920	4,787,646	5,039,080	4,872,376	7,897,194	3,385,568
N90	310,628	2,323,203	3,649,802	3,759,476	4,504,137	346,205
unique proteins	175	146	1158	968	1294	1611
Pfam domains	16,078	16,132	13,573	13,598	13,853	16,048
GO terms (molecular function)	2401	2402	2429	2451	2516	2384
GO terms (biological process)	1794	1898	1977	1994	1973	1830
GO terms (cellular component)	1507	1644	1736	1765	1754	1584

**Figure 2:**
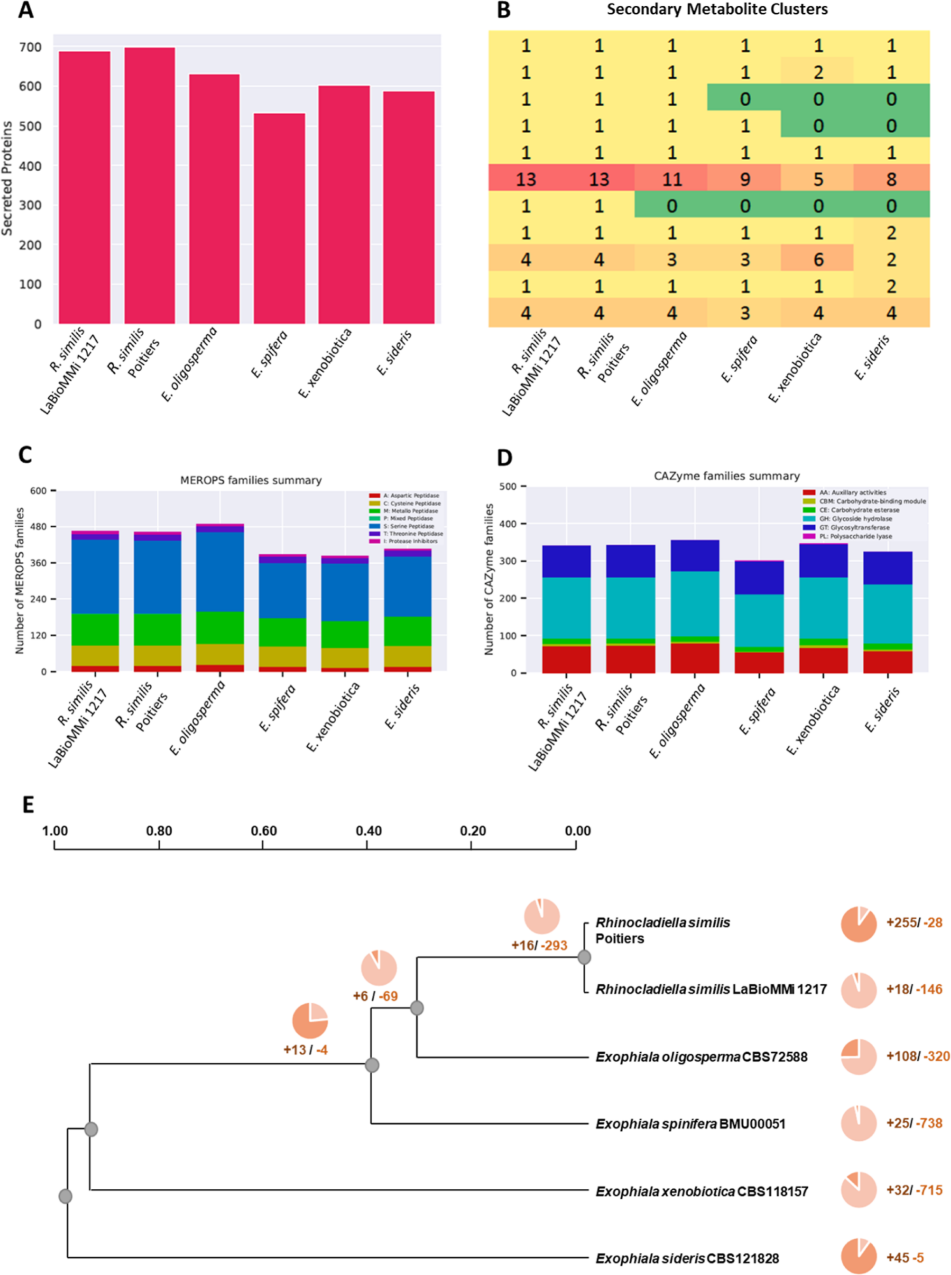
Comparative genomic analysis of *Rhinocladiella similis* LaBioMMi 1217 and its closely related strains. (A) Relative predicted amount of secreted proteins. (B) Predicted biosynthetic gene clusters. (C) Relative distribution of the predicted protease (MEROPS) groups. (D) Bar plot of the CAZyme family domains of *Rhinocladiella* and *Exophiala* species. (E) Phylogenetic tree showing gain and loss of InterPro domains among six genomes (*R. similis*, *n* = 2; *Exophiala* spp., *n* = 4).

Comparison of the two *R. similis* genomes with the closely related strains belonging to the *Exophiala* genus revealed several distinctive characteristics. There was a strong positive correlation between genome size and the number of genes (*R* = 0.9319, *p* < 0.01). The species with the largest genome size and gene content was *E. oligosperma*, followed by *R. similis* species. Both were phylogenetically related, indicating that gene expansion occurred in both species (Table 1), as reflected by a prominent expansion of Pfam domains. Overall, this pattern was also observed in secreted proteins (Figure 2A), secondary metabolite clusters (Figure 2B), and proteases (MEROPS, Figure 2C) but not in carbohydrate-degrading enzymes (CAZymes), particularly when compared with the pectin lyase genes of *E. spinifera* and *Exophiala xenobiotica* (Figure 2D). Notably, the MEROPS subfamilies A1A, S33, S12, S09X, M20D, and C69 were expanded in the *R. similis*/*E. oligosperma* dyad relative to other *Exophiala* species (Figure 2C). Similarly, we also observed an expansion of secondary metabolite clusters related to the production of isocyanide nonribosomal peptide (NRP) and other NRPs in those species (Figure 2B). Regarding the CAZyme families, we observed an expansion of glycoside hydrolases (GHs) GH3, GH32, and GH130 and auxiliary activities (AAs) AA1, AA7, and AA8 (Figure 2D). Finally, the transcription factor classes fungal Zn^2^-Cys^6^ binuclear domain (IPR001138) and KilA-N domain (IPR018004) were expanded in the *R. similis*/*E. oligosperma* dyad, whereas the helix–loop–helix DNA-binding domain (IPR011598) and STE-like transcription factor (IPR003120) were contracted (Supporting Information Figure S1). These genomic differences might impact the species’ ecology. Besides these differences, we observed a negative correlation between the number of proteins and the GO terms related to biological processes (*R* = −0.9003, *p* < 0.05) and cellular components (*R* = −0.8617, *p* < 0.05), indicating that species with fewer protein-coding genes had a more diversified metabolism compared with species with a higher number of protein-coding genes.

We used the CAFE5 tool to test the distribution of such gene families using the birth–death model. *R. similis* LaBioMMi 1217 exhibited an expansion of 18 and a reduction of 146 InterPro domains (*p* < 0.05; Figure 2E). The GO term conversion of the acquired InterPro domains revealed that they were related to metabolic processes associated with aromatic and nitrogenous compounds and organic acid transport. In contrast, the contracted GO terms were related to the regulation of transmembrane transport, DNA-templated transcription, catalytic processes, protein transport, and transcription regulation from RNA polymerase II promoter. These findings indicated that *R. similis* LaBioMMi 1217 lost domains related to transcription control as well as protein degradation, folding, and transport.

**Figure 3:**
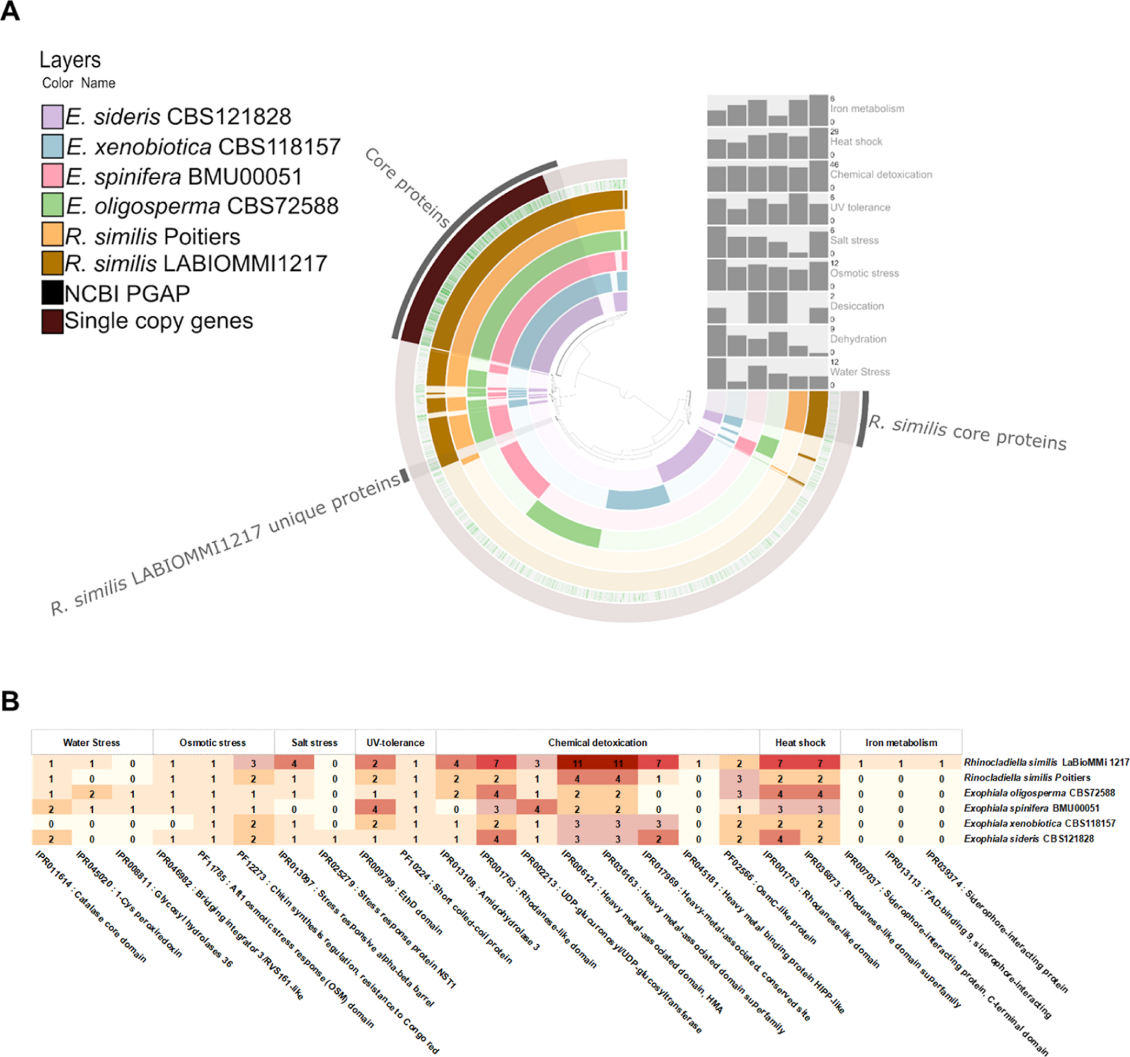
(A) Comparative analysis of the *Rhinocladiella* and *Exophiala* genomes illustrating the shared and unique proteins. The gray bars indicate the annotated protein counts associated with specific functions. (B) Relative distribution of predicted protein terms in the Pfam and InterPro databases filtered according to dehydration, desiccation, rehydration, chemical stress, UV resistance, and iron metabolism.

We observed similarities and differences between the studied strains in the pangenome generated from the annotated genomes. We identified 117 unique predicted proteins and 972 core proteins in the *R. similis* LaBioMMi 1217 genome. Additionally, compared with the other analyzed strains, *R. similis* LaBioMMi 1217 genes related to proteins potentially useful in astrobiologically relevant conditions were more evident. Therefore, we investigated the metabolic adaptations of *Exophiala* and *Rhinocladiella* that might facilitate their viability under challenging environmental conditions similar to those on Mars by analyzing Pfam and InterPro terms associated with dehydration, desiccation, rehydration, heat shock, chemical detoxification, osmotic stress, UV resistance, and Fe metabolism (Figure 3A). The comparative analysis also revealed that *R. similis* LaBioMMi 1217 had higher number of proteins related to Fe metabolism (*n* = 6), heat shock (*n* = 29), and chemical detoxification (*n* = 46). Notably, *R. similis* LaBioMMi 1217 also had higher number of proteins related to UV tolerance (*n* = 4), salt stress (*n* = 5), and osmotic stress (*n* = 11) but lower number of proteins related to desiccation (*n* = 1), dehydration (*n* = 1), and water stress (*n* = 5) compared with the other strains.

Table S2 lists the proteins associated with each function screened in the genomes of the analyzed strains. Of these, 23 were selected for further investigation (Figure 3B), revealing notable differences between the Pfam and InterPro terms among the analyzed species. Proteins associated with dehydration and omostic stress, such as those in the catalase domain (IPR011614), and those associated with osmotic stress response, extracellular FtsH production, and chitin synthesis regulation (PF11785, and PF12273, respectively) were observed in all analyzed strains. When filtered for terms related to saline stress, stress responsive alpha-β barrel protein (IPR013097) was present in all strains, except for *E. spinifera*. Additionally, *R. similis* LaBioMMi 1217 possessed multiple genes related to the synthesis of proteins involved in heavy metal detoxification (IPR045181, and IPR017969), siderophore production siderophore and transport (IPR007037, IPR013113, and IPR039374).

### 3.3 Effect of MGS-1 on *R. similis* LaBioMMi 1217 Morphology

**Figure 4:**
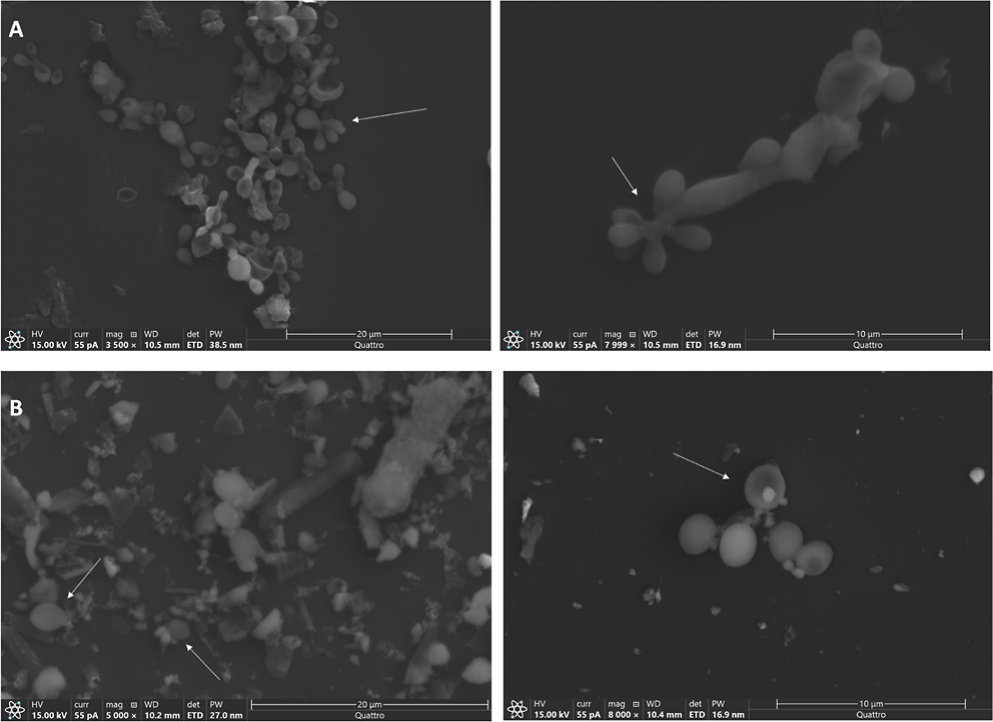
Scanning electron microscopy images of *R. similis* LaBioMMi 1227. *Rhinocladiella similis* LaBioMMi 1227 cultured in (A) potato dextrose broth (PDB) and (B) PDB supplemented with MGS-1 at a ratio of 1:4 (v/w). White arrows indicate cellular structures (pseudohyphae and yeast forms).

LaBioMMi 1217 was cultured in potato dextrose broth, with and without the addition of synthetic Martian regolith MGS-1, and morphological changes were observed using scanning electron microscopy SEM. Fungal pseudohyphae were observed in the absence of MGS-1 (Figure 4A), indicating a transition between unicellular (yeast-like) and multicellular (filamentous) phases characteristic of dimorphic fungal species. These structures are formed by yeast-like cells that remain attached after cell division and form tube-like chains, but they do not possess the same degree of cohesion and cytoplasmic continuity as genuine hyphae. In the experiment containing MGS-1, the yeast-like form of the fungus, characterized by a unicellular spherical or oval shape, was dispersed among the minerals present in the regolith (Figure 4B).

### 3.4 Effect of MGS-1 on Metabolite Extraction and Experimental Control Design

To determine the most suitable control experiment for statistical comparison, the crude extracts obtained from all samples were subjected to UHPLC–MS/MS, and the results were evaluated using unsupervised analysis (PCA). Supporting Information Figure S2A presents the discrepancies among all groups in the unsupervised analysis, revealing distinctions between blanks and adjusted or unadjusted samples. The box plot in Supporting Information Figure S2B exemplifies the comparison of feature areas *m*/*z* 237.0756 and RT 4.985 for adjusted and unadjusted controls. The increased area in the adjusted control feature (with MGS-1 incorporation during extraction) compared with the unadjusted control indicated that MGS-1 optimized the extraction process. Thus, we chose to proceed with our investigation of the microorganism’s metabolism by comparing the adjusted control samples with those containing fungus with regolith.

### 3.5 Metabolomic Differentiation in *R. similis* LaBioMMi 1217 Cultured with MGS-1

**Figure 5:**
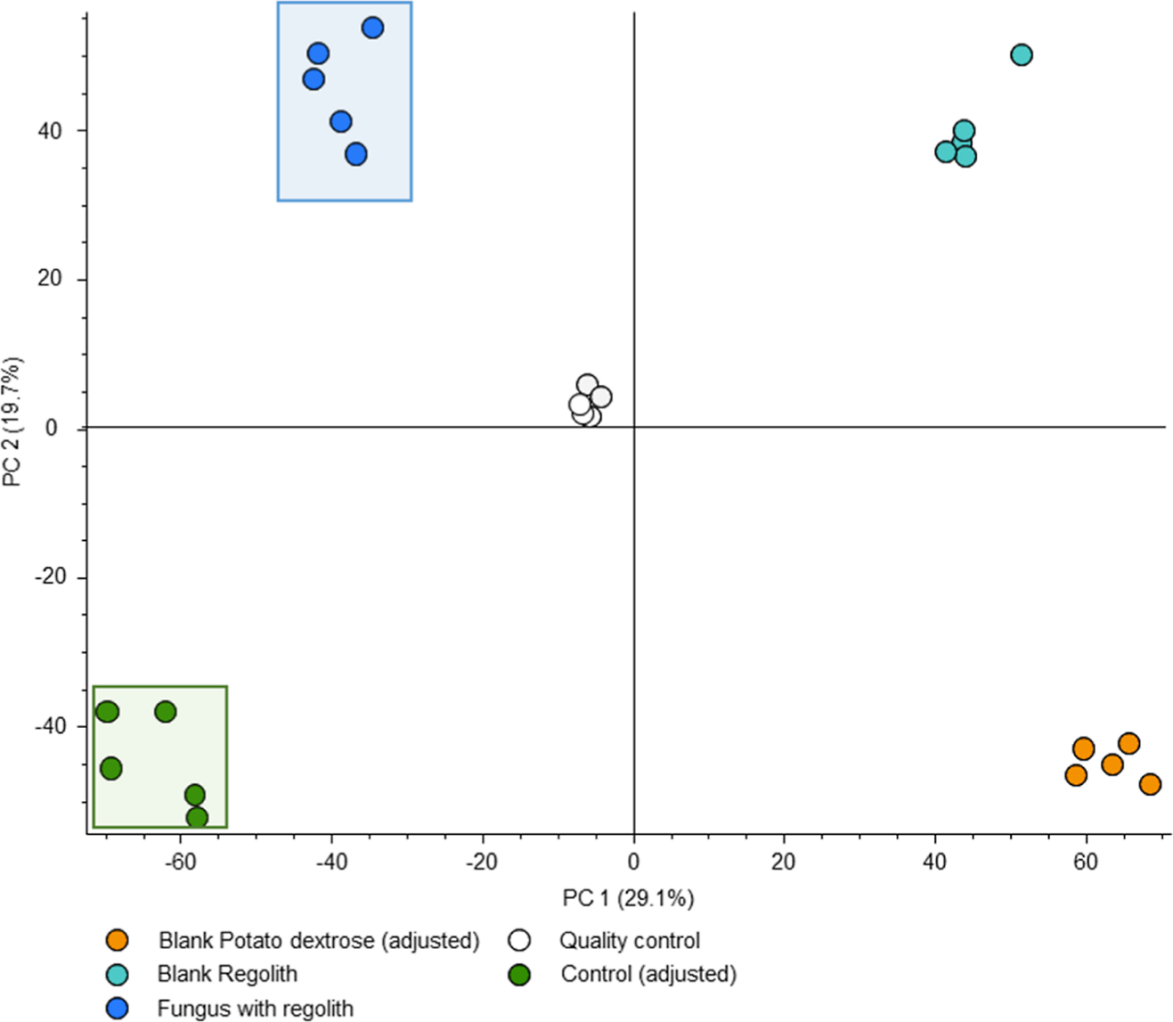
Statistical analysis of UHPLC–MS/MS data of *R. similis* LaBioMMi 1217 in MGS-1 regolith medium and under control conditions. Principal component analysis plot showing variances in metabolite production between control and regolith medium experiments and clustering among replicate samples.

To investigate the effect of the synthetic Martian regolith MGS-1 on *R. similis* metabolism, we used UHPLC–MS/MS to evaluate the crude extracts of the fungus in the presence of the control regolith and its respective blanks. PCA was conducted to provide an overview of the data. The PCA score plot (Figure 5) revealed that the first two principal components accounted for 48.8% (29.1% and 19.7% for PC1 for PC2, respectively) of the overall data variance, showing a separation trend between the fungus in the presence of regolith (fungus with regolith) and the fungus cultured solely in PDB (adjusted control). The control group samples were distributed throughout the third quadrant, and the distribution of the samples of the fungus with regolith varied in the first quadrant. Because the separation between the groups was evident using the unsupervised method, a supervised analysis was unnecessary. The reproducibility of the instrumental system (i.e., both the chromatographic and mass spectrometric components) was assessed by jointly analyzing the QC samples, which were tightly clustered in the center of the PCA score plot, indicating stable performance (Figure 5).

**Figure 6:**
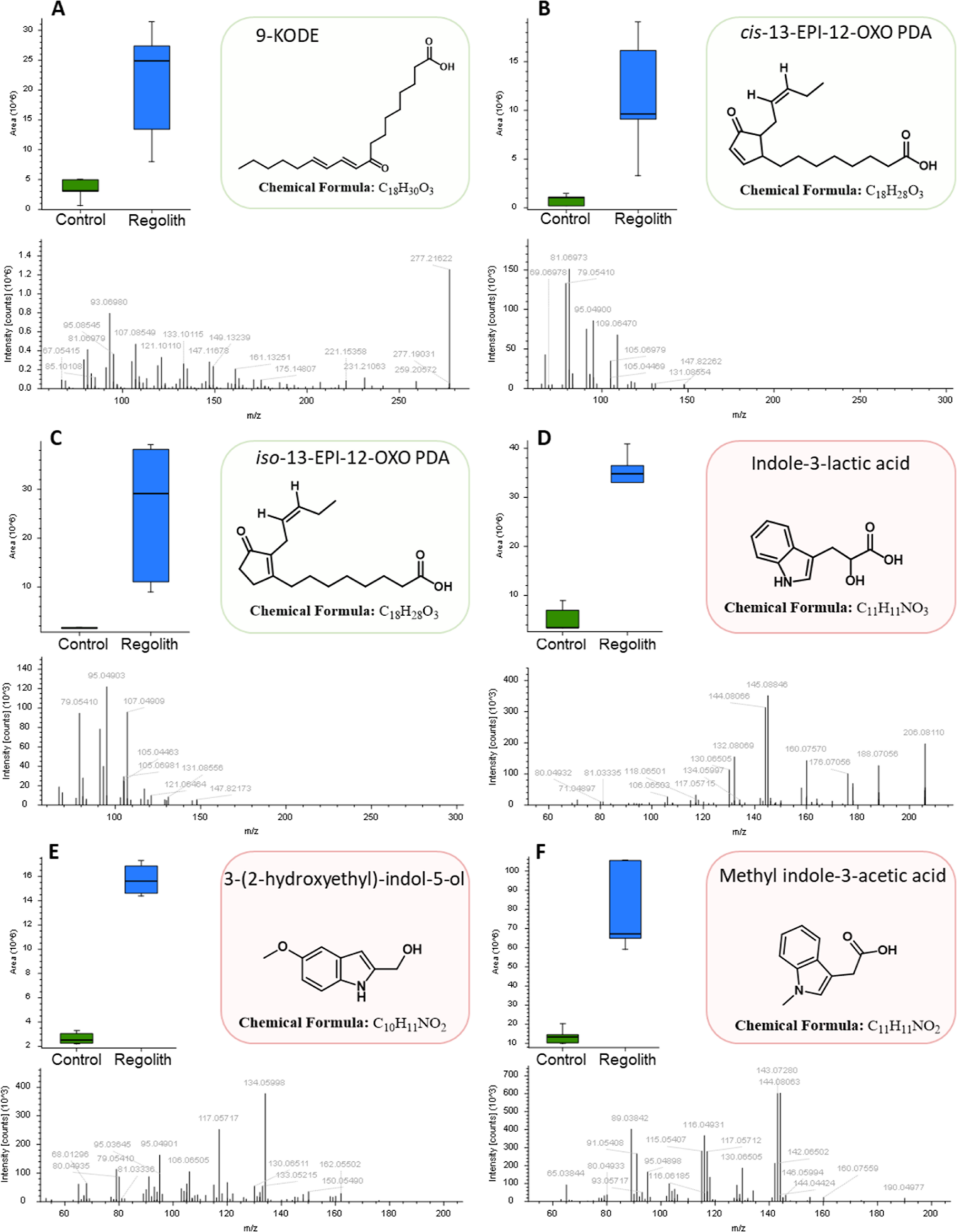
MS/MS spectra of the annotated molecules accompanied by a box plot illustrating the average ion intensity of molecules upregulated by the fungus when cultured in the presence of MGS-1. These include the oxidized fatty acids: (A) 9-KODE, (B) *cis*-13-EPI-12-OXO-PDA, and (C) iso-13-EPI-12-OXO-PDA. Additionally, the indolic derivatives: (D) indole-3-lactic acid, (E) 3-(2-hydroxyethyl)-indole-5-ol, and (F) methyl indole-3-acetic acid.

We considered a log_2_ fold change ≥2 as an indicator of metabolites upregulated in the presence of MGS-1. Such screening yielded 244 features with a significant increase in their area in the presence of MGS-1, indicating that the metabolites were potentially induced by the mineralogical components of MGS-1. Of them, 211 were statistically significant (*t*-test, *p*-value ≤0.05). Six metabolites were annotated based on precise mass analysis (error range: 0–2 ppm) and MS/MS fragmentation. The annotated compounds were identified as 3-(2-hydroxyethyl)-indol-5-ol (*m*/*z* 178.0862, *p*-value = 1.13 × 10^–4^), methyl indole-3-acetic acid (*m*/*z* 190.0862, *p*-value = 3.07 × 10^–3^), indole-3-lactic acid (*m*/*z* 206.0811, *p*-value = 2.61 × 10^–4^), iso-12-oxo-phytodienoic acid (iso-13-EPI-12-OXO-PDA; *m*/*z* 293.2111, *p*-value = 1.05 × 10^–3^), *cis*-12-oxo-phytodienoic acid (*cis*-13-EPI-12-OXO-PDA; *m*/*z* 293.2111, *p*-value = 8.59 × 10^–4^), and 9-oxo-10,12-octadecadienoic acid (9-KODE; *m*/*z* 295.22681, *p*-value = 2.22 × 10^–2^). 9-KODE was classified as a Level 1 annotation because its MS/MS spectrum and RT were comparable with an analytical standard. Figure 6 shows the MS/MS spectra and box plots of the areas of putatively annotated metabolites.

The MS/MS spectra of oxidized fatty acids, such as iso-13-EPI-12-OXO-PDA (*m*/*z* 293.2111), *cis*-13-EPI-12-OXO-PDA (*m*/*z* 293.2111), and 9-KODE (*m*/*z* 295.2268), exhibited fragmentation profiles in positive-ion mode that appeared to act as a unique signature for each molecule. As illustrated in the spectra presented in Figure 6A–C, although there were similarities in fragment ions, the relative intensities differed significantly. Confidence in the annotations indicated by databases was strengthened by the consistency observed for 9-KODE when compared with its known analytical standard (Supporting Information Figure S8) because it presented the same MS/MS spectrum and similar RT.

We delved deeper into the spectral peculiarities of indolic acid derivatives. The MS/MS spectrum (Figure 6D) of indole-3-lactic acid with [M + H]^+^ at *m*/*z* 206.0811 highlighted a base peak at *m*/*z* 145.0884. This fragmentation was characteristic of the indole core after losing an acid group, forming a stabilized cation. A high-intensity peak was also observed at *m*/*z* 144.0806, which was interpreted as the additional loss of an H atom from the ion at *m*/*z* 145.0884. The ion at *m*/*z* 188.0705, representing a mass difference of 18 Da relative to the molecular ion, indicated the loss of H_2_O. The peak at *m*/*z* 160.0757 indicated the loss of the carboxyl group (−COOH). Peaks at *m*/*z* 132.0806 and *m*/*z* 130.0650 indicated subsequent fragmentations of the indole structure, representing alternative decomposition pathways during fragmentation.

The MS/MS spectrum (Figure 6E) of 3-(2-hydroxyethyl)-indole-5-ol with [M + H]^+^ at *m*/*z* 178.0862 displayed main fragments at *m*/*z* 134.0559, *m*/*z* 117.0517, *m*/*z* 106.0605, and *m*/*z* 95.0491, indicating indole ring fragmentation and subsequent loss of functional groups. A base peak was also observed at *m*/*z* 134.0559, indicating a neutral loss of the hydroxyethyl group [M + H – C_2_H_4_O]^+^, representing the most stable fragment formed during ionization.

The MS/MS spectrum (Figure 6F) of methyl indole-3-acetic acid with [M + H]^+^ at *m*/*z* 190.0862 showed a base peak at *m*/*z* 144.0806, characteristic of indole nucleus fragmentation after losing the methoxy group (−OCH_3_). Another high-intensity peak was observed at *m*/*z* 143.0728, indicating the simultaneous fragmentation of the indole core and the loss of an H atom. Additional fragment ions included *m*/*z* 160.0756, possibly related to the loss of the carboxyl group (−COOH); *m*/*z* 116.0493 and *m*/*z* 117.0512, which may represent subsequent indole nucleus fragmentations; and peaks at *m*/*z* 95.0498 and *m*/*z* 89.0384, representing more extensive structural fragmentations.

**Figure 7:**
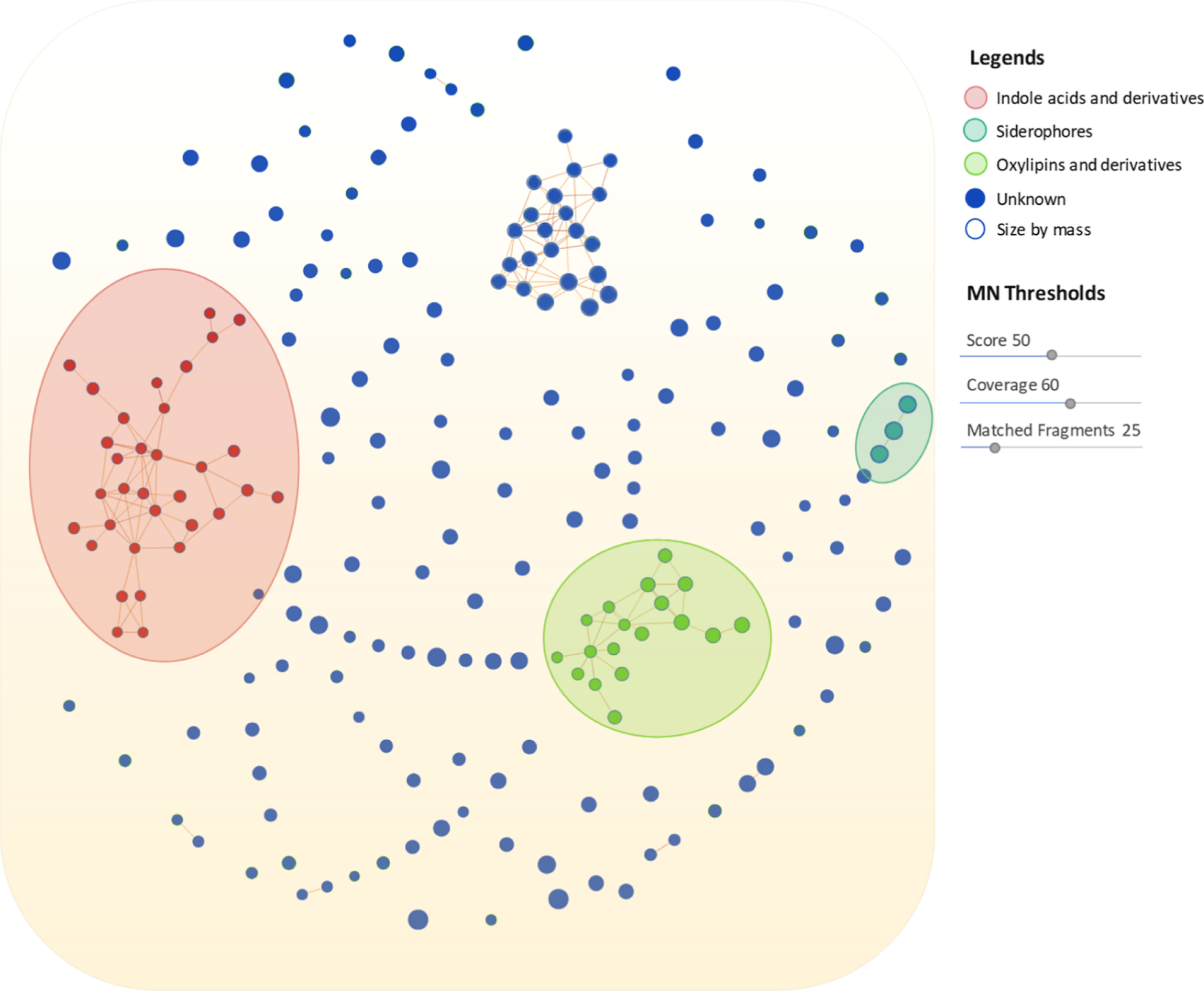
Molecular network generated using Compound Discover v3.3 software. Here, each node represents a feature with high-resolution mass and retention time. The software also grouped metabolites according to their structural functions. Molecular families, including indole derivatives, oxylipins, and siderophores, were classified based on the annotated nodes.

To determine whether other features induced by MGS-1 were related and to potentially propagate annotation to other ions, we performed molecular network analysis using the spectral comparison-based tool CD v3.3 with the MS/MS data of all 211 statistically significant features. The spectra were grouped based on coverage, the number of matching fragments, and similarity scores, resulting in clustering into four spectral families (Figure 7), indicating that MGS-1 increased molecule production and induced entire molecular families.

**Figure 8:**
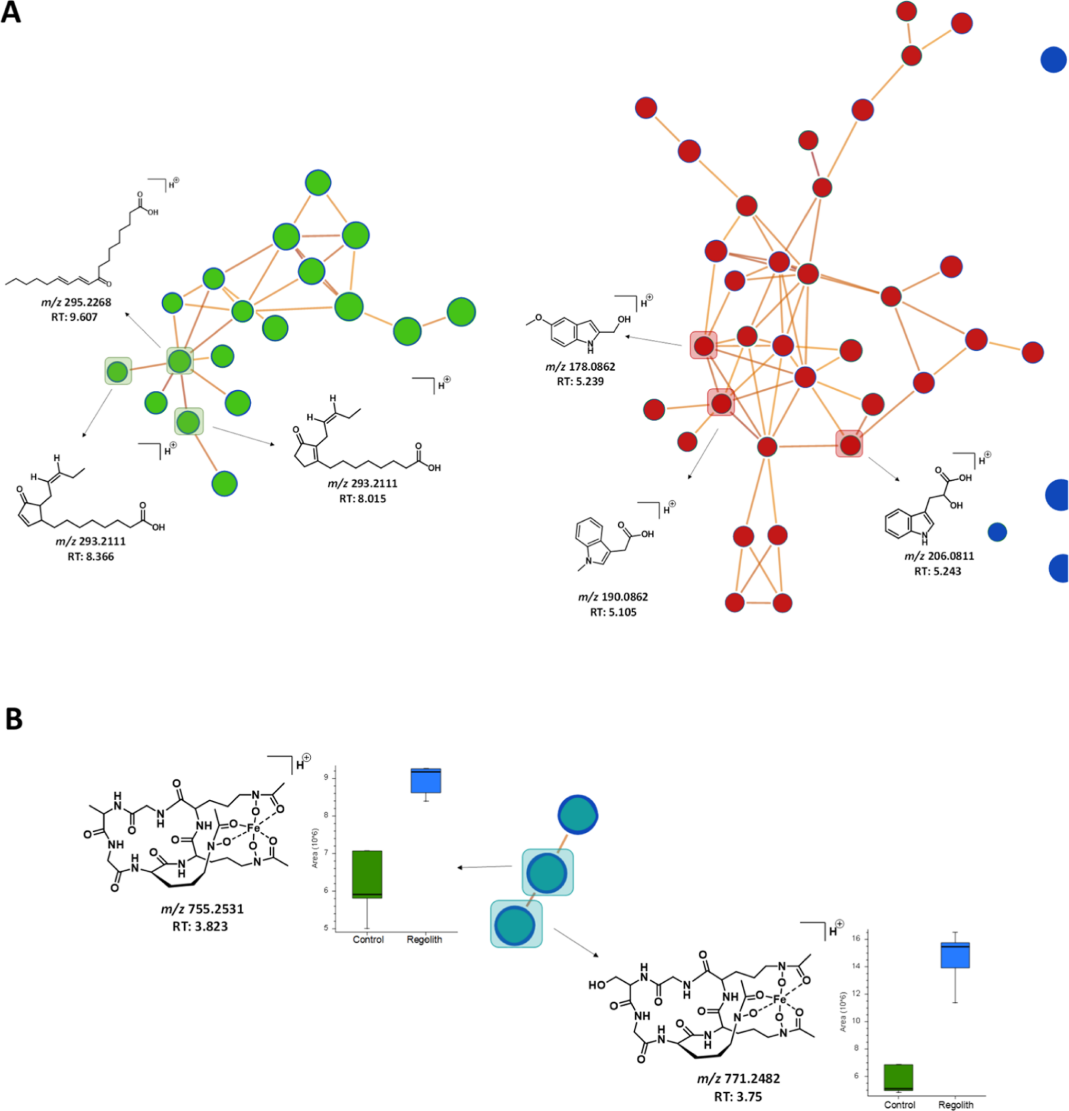
Expansion of molecular clusters according to the structure of their respective ions. (A) Molecular network (MN) of the two annotated families, as obtained by MS/MS spectrum comparison. Green, indolic acid and its derivatives; red, oxylipins and derivatives. (B) MN of the siderophore molecular family. Box plots of areas with increased production in the MGS-1 regolith experiment.

The previously annotated compounds were distributed into two molecular families: indolic acid derivatives and oxylipin derivatives (Figure 8A).

Based on manual observation of the MS/MS spectra, a molecular family comprising only three nodes was annotated. This was prompted by an initial observation of a difference of 18 Da between the ion at *m*/*z* 755.2531 and the ion at *m*/*z* 771.2482, indicating a difference in hydroxylation. We also observed an atypical isotopic pattern of the precursor ions (Supporting Information Figure S11), indicating the presence of an Fe atom. Using fragmentation mechanisms, these two features were manually annotated as siderophores and were supported by the literature.^57^ Supporting Information Figures S9 and S10 present proposed fragmentation mechanisms for these molecules. Thus, the ions at *m*/*z* 755.2531 (*p*-value = 5.13 × 10^–3^) and *m*/*z* 771.2482 (*p*-value = 3.91 × 10^–2^) were putatively annotated (Level 2) as ferrichrome C and ferricrocin, respectively. Figure 8B presents the molecular family of siderophores, and the box plots of the areas show the increased production of these two molecules in the presence of synthetic Martian regolith MGS-1. All *m*/*z* values of the nodes present in the families annotated by the molecular network were organized and can be found in Table S3 of the Supporting Information.

### 3.6 Detectability of Biosignatures Produced by *R. similis* LaBioMMi 1217 in Synthetic Martian Regolith Matrices Using LDI-MS

**Figure 9:**
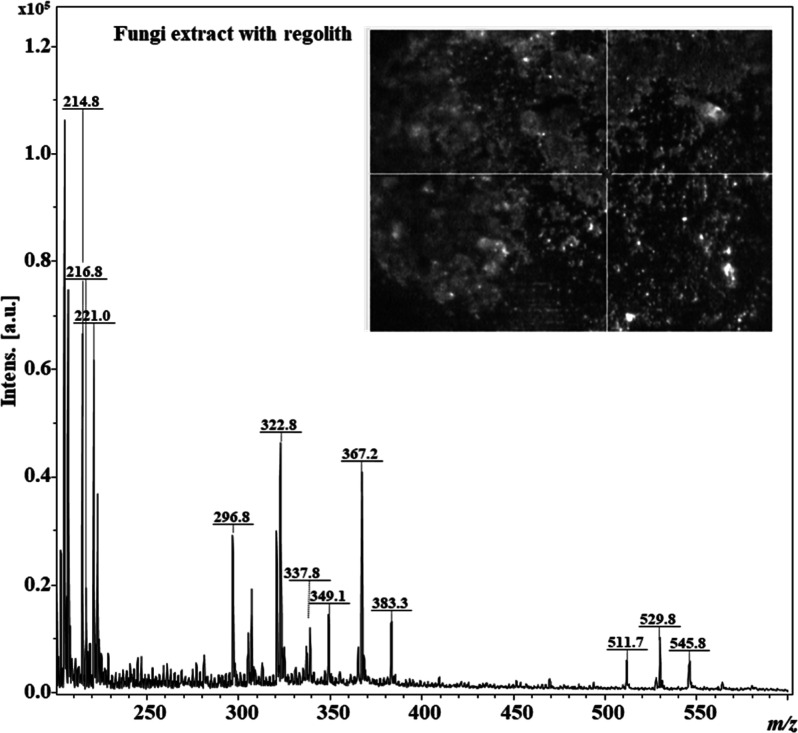
Mass spectrometry spectrum obtained by laser desorption ionization in the *m*/*z* 200–600 range for the extract of *Rhinocladiella similis* LaBioMMi 1217-doped MGS-1.

To investigate the capability of LDI-MS to detect biosignatures on Mars, the extract containing the metabolites identified as biomarkers of the fungus–mineral interaction in the untargeted metabolomics experiment underwent a simulation of detection by LDI-MS. Measurements in the range of *m*/*z* 200–600 were performed for samples containing the extract of *R. similis* LaBioMMi 1217 cultured in the presence of MGS-1 and dispersed in the same regolith. Despite the roughness of the MGS-1 matrix, we were able to obtain spectra with sufficient mass resolution to identify the target compound. We observed a variety of high-intensity ions in the *m*/*z* 200–250 range and other ions with lower intensity in the *m*/*z* 300–350 range. Some ions were also observed *m*/*z* 510–550 range, indicating the presence of organic molecules with lipid-like characteristics. No ions were detected in the mass region of siderophores. Figure 9 presents the expanded spectrum in the *m*/*z* 200–600 range.

## 4 Discussion

The search for eukaryotic models, such as fungi, in the field of astrobiology, is gaining attention due to their significant contributions in extreme environments on Earth, indicating that they are capable of potential contributions on other planets.^14^ The fungus *Cryomyces antarcticus*, a black yeast isolated from the McMurdo dry valleys in Antarctica, is recognized as the most stress-resistant fungal microorganism found on Earth and in simulated space environments, and it has been used as a model for life on Mars.^58^ Our results indicated that in addition to the genus *Cryomyces*, representatives of the family *Herpotrichiellaceae* also show potential astrobiological applications because some are polyextremophiles, capable of surviving under a wide range of stressors. Thus, they may be capable of withstanding the extreme conditions of extraterrestrial environments.^59−61^ Our phylogenomic and metabolomic findings contribute to understanding these microorganisms as candidate models of astrobiological life, with a primary focus on their biosignature production and interaction with the geochemical aspects of Mars. To our knowledge, this is the first report to explore *R. similis* as an astrobiological model and focus on its extremophilic genomic traits and biosynthesized metabolites.

The genetic characterization of isolate LaBioMMi 1217 revealed that in addition to being a strain of the species *R. similis*, it was closely related to yeasts of the genus *Exophiala*, such as *E. oligosperma*. Some members of the genus *Exophiala* have been studied for astrobiological purposes. For example, *Exophiala* sp. strain 15LV1, isolated from the Atacama Desert, survived high levels of UV radiation (exposure to 1 kJ/m^2^ of UVC) and stratospheric balloon flight experiments.^59^

Our genomic analysis revealed that species of *Rhinocladiella* and *Exophiala* produced predicted proteins (Pfam and InterPro terms) associated with dehydration and desiccation stress responses. Genetic characteristics associated with desiccation and oligotrophy were observed in *R. similis* LaBioMMi 1217 and *E. oligosperma* CBS72588, which might promote their adaptation to conditions with low nutrient levels and limited organic matter, making them good candidates for experiments simulating the chemical conditions of Martian soil.^62^ All analyzed strains produced proteins predicted as related to UV resistance. Because of their highly pigmented nature, the presence of laccases might be associated with melanin biosynthesis.^63^

Regarding geochemistry, Martian regoliths are as rich in ferric minerals (i.e., hematite) and perchlorate salts. Thus, by filtering for proteins associated with chemical detoxification and Fe metabolism, we found that when compared with the other analyzed strains, *R. similis* LaBioMMi 1217 produced a considerable number of proteins that could be used as mechanisms to assess environmental stress, such as the 74 proteins associated with the cytochrome P450 superfamily. Studies on the bacterial genus *Rhodococcus* have demonstrated significant overexpression of these proteins when cultured in the presence of perchlorate.^64^ Additionally, our strain (*R. similis* LaBioMMi 1217) was the only one to produce proteins associated with siderophore transport, indicating its capability to survive in environments rich in perchlorate, such as Martian brines. We also observed that *R. similis* LaBioMMi 1217 was the only strain to produce proteins associated with the production and transport of siderophore molecules. Considering the conditions on Mars, these proteins may play a role in the mechanism of Fe acquisition from the oxides present on Mars.^65,66^

The surface of Mars is an extremely hostile environment for all forms of life, mainly due to the combination of UV radiation and perchlorates in the regolith. Hence, the subsurface has emerged as an interesting niche to investigate how astrobiological models cope in these environments. To better understand the microorganism–regolith interactions from a Martian perspective, and considering the capabilities of *R. similis*, we investigated its morphology and metabolism using experiments simulating Martian geochemistry. Our investigation of morphological changes in *R. similis* LaBioMMi 1217 when interacting with simulated Martian soil revealed that it used its dimorphic capacity to survive in these harsh conditions. This same type of adaptation was also observed when the same yeast was cultured in perchlorate brines,^67^ indicating that this morphological change may be related to the presence of saline and oxidative stress in the simulated soil medium. In the presence of environmental stress, this same plasticity has been reported for several other melanized fungi.^68^ Given the high complexity of the synthetic regolith used in our experiments and the addition of perchlorate, it is difficult to ascertain which factor is truly responsible for this change in plasticity because the presence of regolith alone plays an oxidizing role due to its high hematite content.^55,69^

Molecules were also induced by the Martian geochemistry. These interesting molecular classes revealed insights into how *R. similis* LaBioMMi 1217 metabolism might change under Martian conditions. The observation of oxylipins, which are metabolites derived from polyunsaturated fatty acids, such as arachidonic acid, as a biomarker of the fungus–Martian geochemical interaction is quite remarkable. In complex organisms, oxylipins act as signaling molecules that regulate developmental processes and mediate responses to biotic and abiotic stressors.^70^ Although the function of this class of molecules in fungi remains poorly understood, experiments with filamentous fungi of the *Aspergillus* genus have demonstrated that oxylipins are crucial mediators of the stress response to aid growth, reproduction, and sporulation.^71^ Oxylipin production in yeasts, such as *Candida albicans*, is associated with biofilm formation, which enhances substrate adhesion.^72^ Considering the increased production of these metabolites by the fungus *R. similis* and the predominance of yeast-like cells in the presence of medium containing synthetic Martian regolith, we assume that oxylipins are associated with the change from the filamentous to the yeast-like stage or with biofilm formation to improve contact with regolith particles.

In our experiment, we also observed an increased production of molecules containing the indole group, such as indole lactic acid, which is a key intermediate in melanin biosynthesis in organisms, including black yeasts.^73,74^ Melanin production plays crucial roles in different biological systems, including protection against environmental stressors, such as UV and ionizing radiation, oxidative agents, thermal extremes, and osmotic pressure. For example, experiments with γ radiation led to a significant increase in melanin production by *Exophiala dermatitidis* ATCC 34100,^75^ and the yeast strain *Hortaea werneckii* EXF 225 began to produce melanin to cope with a high salt content.^76^ Because perchlorate was present in the Martian soil mimic used in our experiments, we inferred that increased melanin biosynthesis may be a coping mechanism for osmotic stress. As observed in a study with the same strain under conditions of perchlorate brine, *R. similis* LaBioMMi 1217 showed modulated pigmentation in the presence of perchlorate brines.^67^

Siderophores are secondary metabolites that capture Fe from the environment and are produced by various microorganisms, including fungi.^77^ Fe is an essential micronutrient for most life forms, and the ability to acquire Fe^3+^ from environments where this form is scarce or unavailable is a crucial survival strategy. Among the siderophores, ferricrocin and ferrichrome production by several fungus species, particularly black yeasts, are the most well-known mechanisms of virulence.^78^ The discovery that the fungus *R. similis* LaBioMMi 1217 is capable of increasing ferricrocin production, especially in an Fe-rich environment, represents an intriguing and potentially relevant discovery for astrobiology. The ability to produce ferricrocin derivatives may enable the fungus to access Fe from the regolith that may not be readily available in a form that the fungus can use, such as iron oxide. Additionally, this discovery could have implications for the detection of life on Mars. Because this compound class has a specific chemical complexity and is only produced by living organisms, it may be a potential biosignature.

Regarding the detectability of biosignatures on Mars, the Rosalind Frankin rover, which is being sent on the ExoMars mission by the European Space Agency to search for traces of life, will carry an LDI-MS-type spectrometer. To simulate the molecules induced by Martian regolith, we analyzed the crude extract containing MGS-1-induced molecules using LDI-MS, with the regolith itself serving as the matrix. The LDI-MS spectrum showed a grouping of ions in the *m*/*z* 300 region, which, in previous studies on the same fungal strain using this technique, was associated with the presence of lipids, such as the *m*/*z* 337.8 ion, a possible Fe^2+^ adduct of oleic acid.^20^ Taking into account the high concentrations of Fe in the minerals found in Martian soil,^55^ the *m*/*z* 349.1 ions warrant attention, considering the same chelation mechanism with Fe, likely corresponding to the Fe adduct of the oxylipin 9-KODE, with a theoretical molecular formula of C_18_H_29_O_3_, calc. as [M – H + ^56^Fe^2+^]^+^ at *m*/*z* 349.1, and *m*/*z* 367.2, probably corresponding to the Fe adduct of some oxidized fatty acid, with a theoretical molecular formula of C_18_H_31_O_4_, calc. as [M – H + ^56^Fe^2+^]^+^ at *m*/*z* 367.2.

In terms of astrobiology, the potential for fungi to produce oxylipins in response to regolith interaction means that they can be valuable biosignatures. Furthermore, owing to their lipidic nature, they would be preserved in the Martian environment^79^ for potential detection by LDI-MS in situ in the near future.

## 5 Conclusion

Our research highlights the relevance of *R. similis* LaBioMMi 1217 and other closely related black fungi as robust models for astrobiology due to their adaptations and resilience to extreme environments analogous to those on Mars, particularly those related to Martian geochemistry. Genomic analysis revealed that *Exophiala* spp. and *Rhinocladiella* spp. harbored several stress resistance genes relevant to Martian conditions, including those associated with UV radiation resistance and/or tolerance, cold temperatures, high salinity, and enhanced nutrient uptake in oligotrophic environments. The black fungi model *R. similis* LaBioMMi 1217 exhibited notable morphological plasticity and the ability to produce molecules, such as oxylipins, melanin precursors, and melanin itself, in response to simulated Martian geochemistry. The increased production of oxidized fatty acids, indole derivatives, and siderophores indicates a potential survival strategy under conditions of Martian geochemistry, including the metabolization of Fe from regolith minerals.

The detection of these molecules, particularly those of a lipid nature, such as oxidized fatty acids, indicates their potential as biosignatures of life on Mars. Furthermore, these molecules were detected in simulations using LDI-MS, reinforcing their viability as indicators of life.

## Supporting Information Available

Genomic characteristics of *R. similis* and Exophiala strains; PCA results on the effect of regolith on extraction; heatmaps and orthology analysis of protein family differences; MS/MS spectra and spectral comparisons for bioactive compound identification (PDF)
Interpro/Pfam analysis listing astrobiologically relevant protein families across strains (XLSX)
Mass spectrometry data with molecular clusters, ion formulas, retention times, fold changes, and statistical significance for detected compounds (XLSX)
Accession numbers of conserved genes used for multilocus sequence analysis, including ITS, LSU, RPB, TEF, and TUB gene regions (XLSX)
