# Supplementary material for: Rhinocladiella similis: A Model Eukaryotic Organism for Astrobiological Studies on Microbial Interactions with Martian Soil Analogs
Source: JACS Au. 2024 Dec 23;5(1):187–203. doi: 10.1021/jacsau.4c00869 (PMC11775710; doi:10.1021/jacsau.4c00869)
Supplement: Supplementary file 1 — au4c00869_si_001.pdf [file au4c00869_si_001.pdf]

Supplementary Information

***Rhinocladiella similis*: A Model Eukaryotic Organism for Astrobiological Studies on Microbial Interactions with Martian Soil Analogs**

Alef dos Santos[1,2*], Júnia Schultz[2], Isabella Dal'Rio[2,3], Fluvio Molodon[2,4], Marilia Almeida Trapp[5], Bernardo Guerra Tenório[8], Jason E. Stajich[7], Marcus de Melo Teixeira[8], Eduardo Jorge Pilau[6], Alexandre Soares Rosado[2,9*], Edson Rodrigues-Filho[1*]

[1]Department of Chemistry, Federal University of São Carlos, São Carlos , 13565-905, Brazil

[2]Biological and Environmental Science and Engineering Division (BESE), King Abdullah University of Science and Technology (KAUST), Thuwal, 23955, Saudi Arabia

[3]Paulo de Góes Microbiology Institute, Federal University of Rio de Janeiro, Rio de Janeiro 21941-902, Brazil

[4]Oceanographic Institute, University of São Paulo, São Paulo, 05508-120, Brazil

[5]Analytical Core Lab, King Abdullah University of Science and Technology (KAUST), Thuwal, 23955, Saudi Arabia

[6]Department of Chemistry, State University of Maringá, Maringá, 13565-905, Brazil

[7]Department of Microbiology and Plant Pathology, University of California-Riverside, Riverside, CA, 92521, USA

[8]School of Medicine, University of Brasilia, Brasilia, 70910-900, Brazil

[9]Bioscience Program, Biological and Environmental Science and Engineering Division (BESE), King Abdullah University of Science and Technology (KAUST), Thuwal, 23955, Saudi Arabia

*Corresponding author(s)

Alef dos Santos

alef@estudante.ufscar.br

Alexandre Soares Rosado

alexandre.rosado@kaust.edu.sa

Edson Rodrigues-Filho

edinholabiommi@gmail.com

Table S1. Genomic characteristics of the analyzed strains in this study.

**Species**	***R. similis***	***R. similis***	***E. oligosperma***	***E. spinifera***	***E. xenobiotica***	***E. sideris***
**Strain**	LABIOMMI 1217	Poitiers	CBS72588	BMU00051	CBS118157	CBS121828
**Locus tag**	RBB50	POITI	ExoOl	ExoSp	ExoXe	ExoSi
**Assembly Size**	34,715,284 bp	34,259,553 bp	38,224,514 bp	32,380,025 bp	31,405,760 bp	29,505,589 bp
**Largest Scaffold**	2,943,420 bp	5,475,506 bp	4,470,873 bp	6,251,722 bp	5,554,269 bp	9,941,542 bp
**Average Scaffold**	433,941 bp	2,854,963 bp	267,304 bp	4,625,718 bp	2,093,717 bp	5,901,118 bp
**Number of Scaffolds**	80	12	143	7	15	5
**Scaffold N50**	1,238,920 bp	4,787,646 bp	3,385,568 bp	4,872,376 bp	5,039,080 bp	7,897,194 bp
**GC (%)**	50,98%	50,98%	50,01%	51,92%	51,52%	50,63%
**Number of Genes**	12.908	12.903	13.683	11.405	11.454	11.41
**Number of Proteins**	12.857	12.867	13.646	11.367	11.415	11.385
**Number of tRNA**	51	36	37	38	39	25
**Unique Proteins**	175	146	1.611	968	1.158	1.294
**Proteins with at least 1 ortholog**	12.666	12.71	11.736	10.336	10.15	9.965
**Single-copy orthologs**	5.974	5.974	5.974	5.974	5.974	5.974

Table S2. Interpro/Pfam of astrobiological interest.

Table S3. Features table of mass spectrometry data.

Table S4. Assession number of the conserved genes used for MLSA analysis.

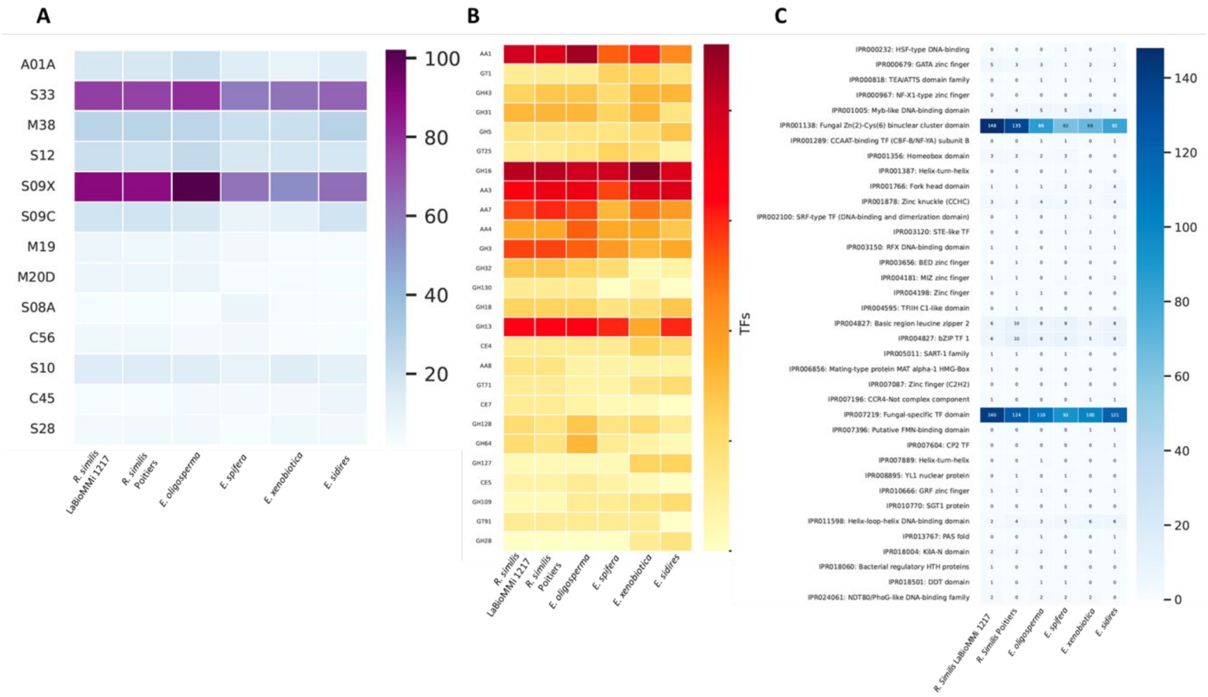

Fig. S1. Heatmaps illustrating protein families that show significant differences between species of the genera *Exophiala* and *Rhinocladiella* used in this study. (A) Merops: Proteases involved in protein degradation processes. (B) CAZymes: Carbohydrate-active enzymes associated with the breakdown and modification of carbohydrates. (C) Transcription Factors: Proteins that regulate the transcription of specific genes.

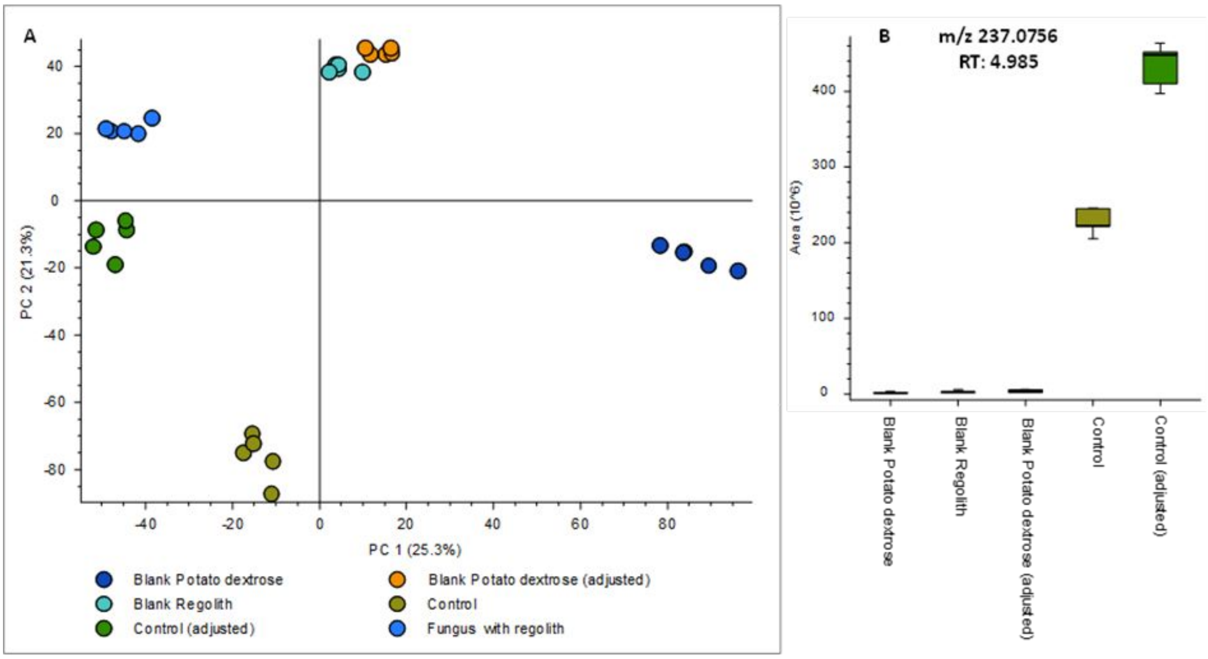

Fig. S2. (A) PCA of all groups, demonstrating the difference caused by adding regolith to the control experiment during the extraction process. **(B)** Illustration of the matrix effect using a Box plot for the areas of the feature with *m/z* 237.0756 RT: 4.985. The extraction of this feature was enhanced by the use of regolith.

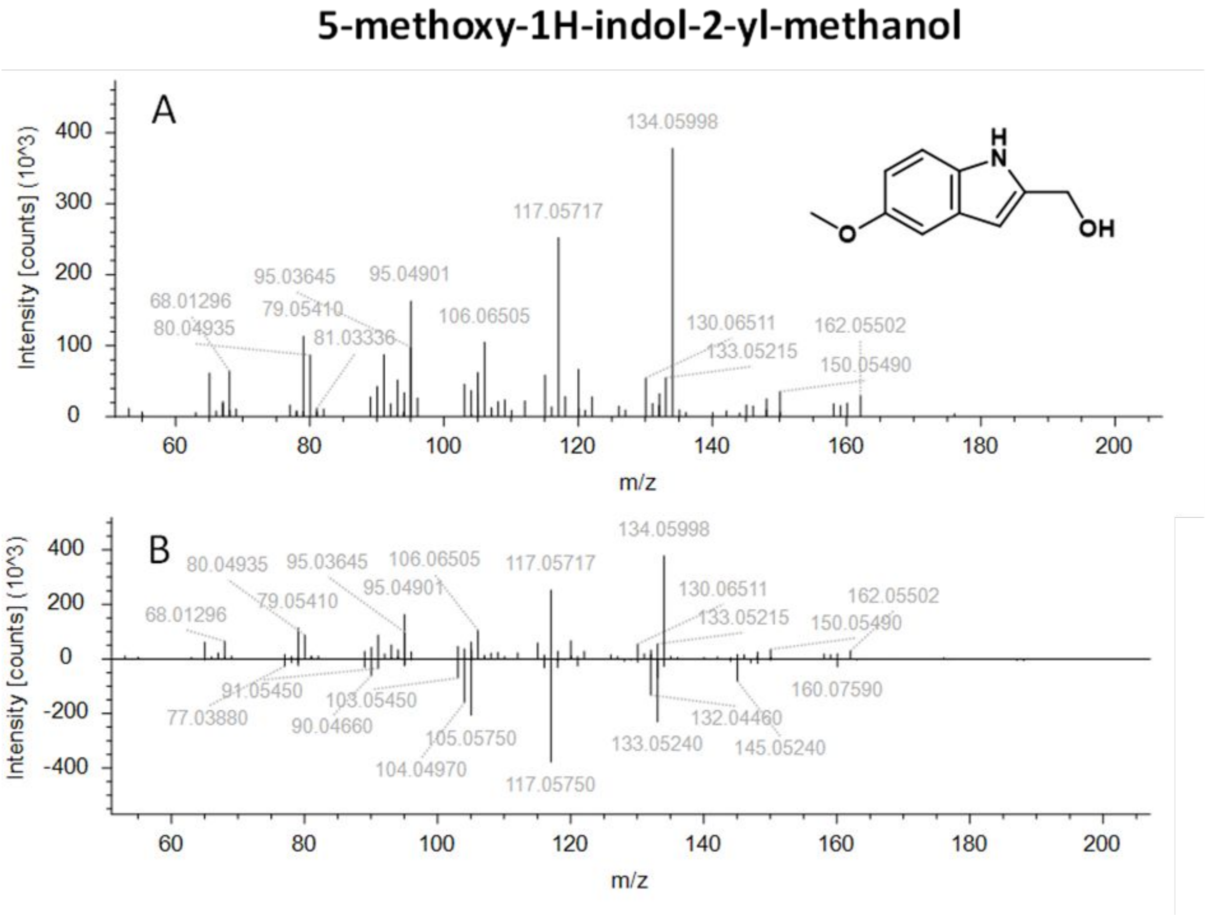

Fig. S3. (A) MS/MS spectrum of the ion with *m/z* 178.08624. **(B)** Spectral comparison between the obtained spectrum and the spectrum of 5-methoxy-1H-indol-2-yl-methanol deposited in the MzVault library.

Methyl indole-3-acetic acid

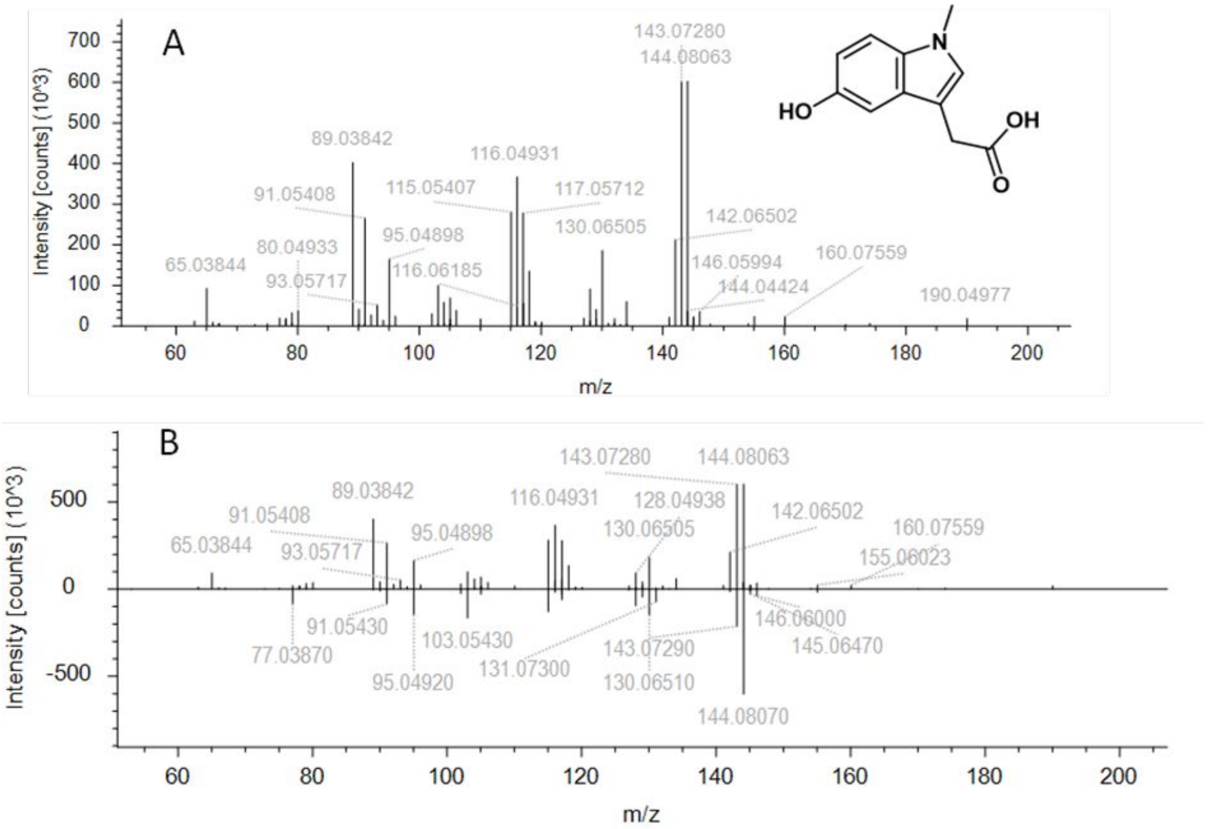

Fig. S4. (A) MS/MS spectrum of the ion with m/z 190.0862. **(B)** Spectral comparison between the obtained spectrum and the spectrum of 5-Methyl indole-3-acetic acid stored in the MzVault library.

Indole-3-lactic acid

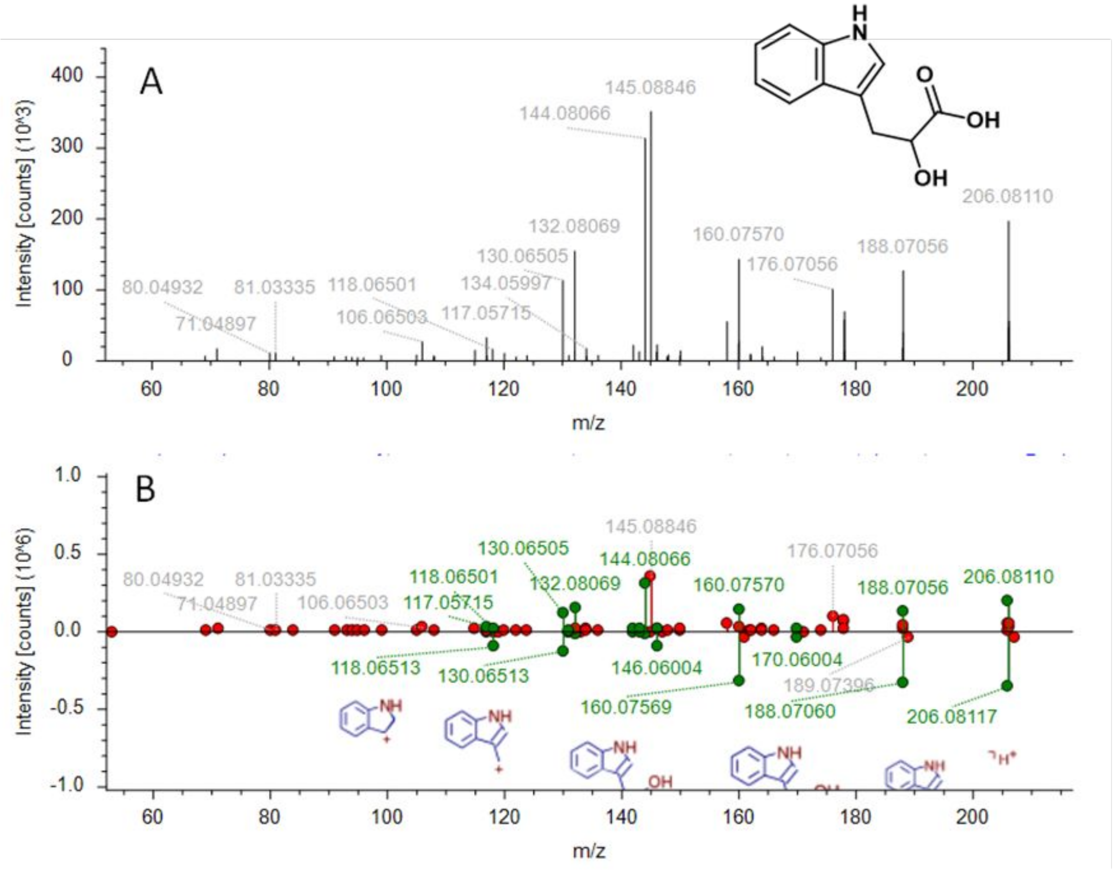

Fig. S5. (A) MS/MS spectrum of the ion with *m/z* 206.8101. (B) Spectral comparison between the obtained spectrum and the spectrum of Indole-3-lactic acid stored in the MZCloud library.

cis-12-Oxo phytodienoic acid

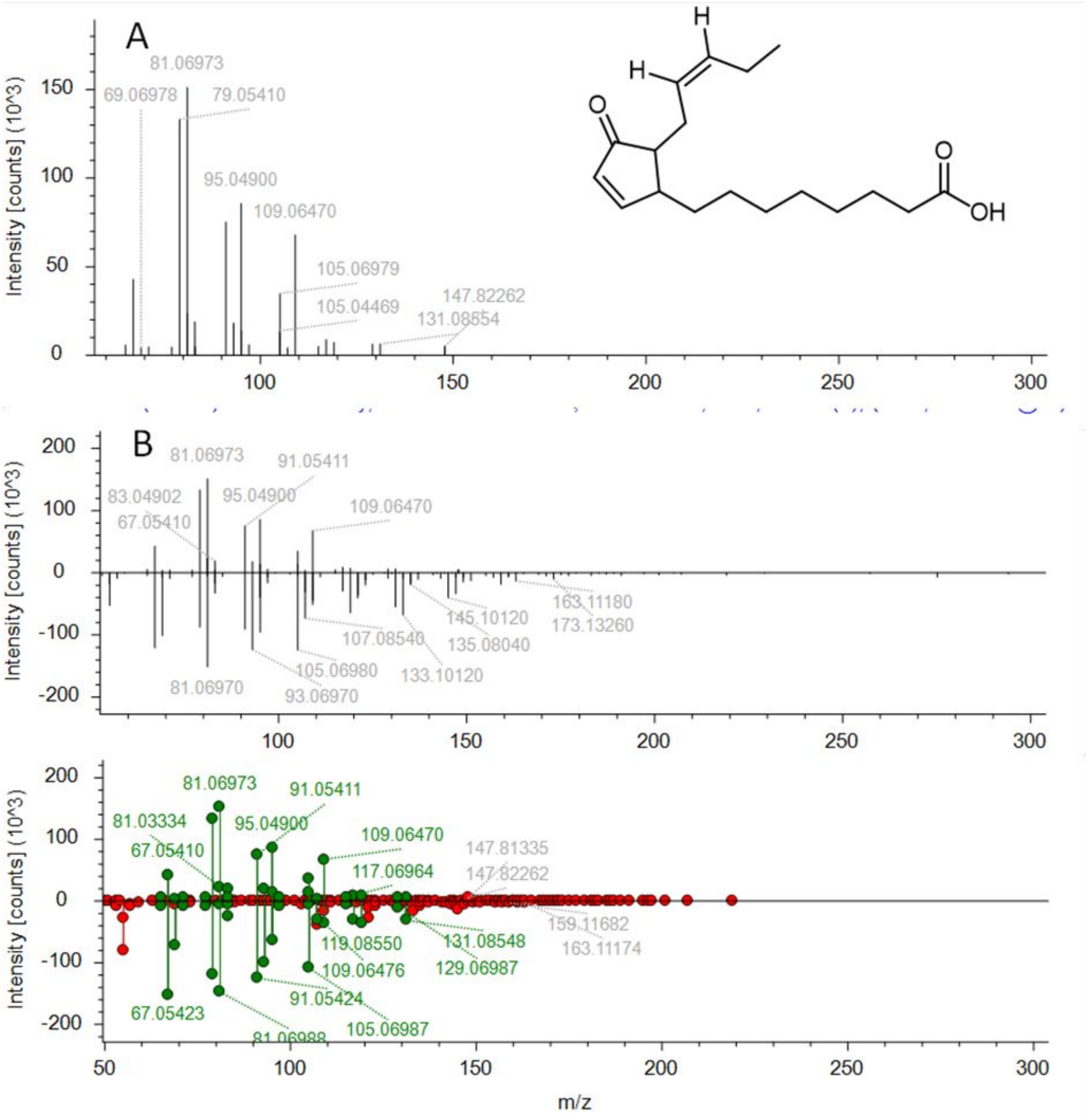

Fig. S6. (**A**) MS/MS spectrum of the ion with *m/z* 293.2111. **(B)** Spectral comparison between the obtained spectrum and the spectrum of cis-12-Oxo phytodienoic acid deposited in the MzVault and MZCloud library.

iso-12-Oxo phytodienoic acid

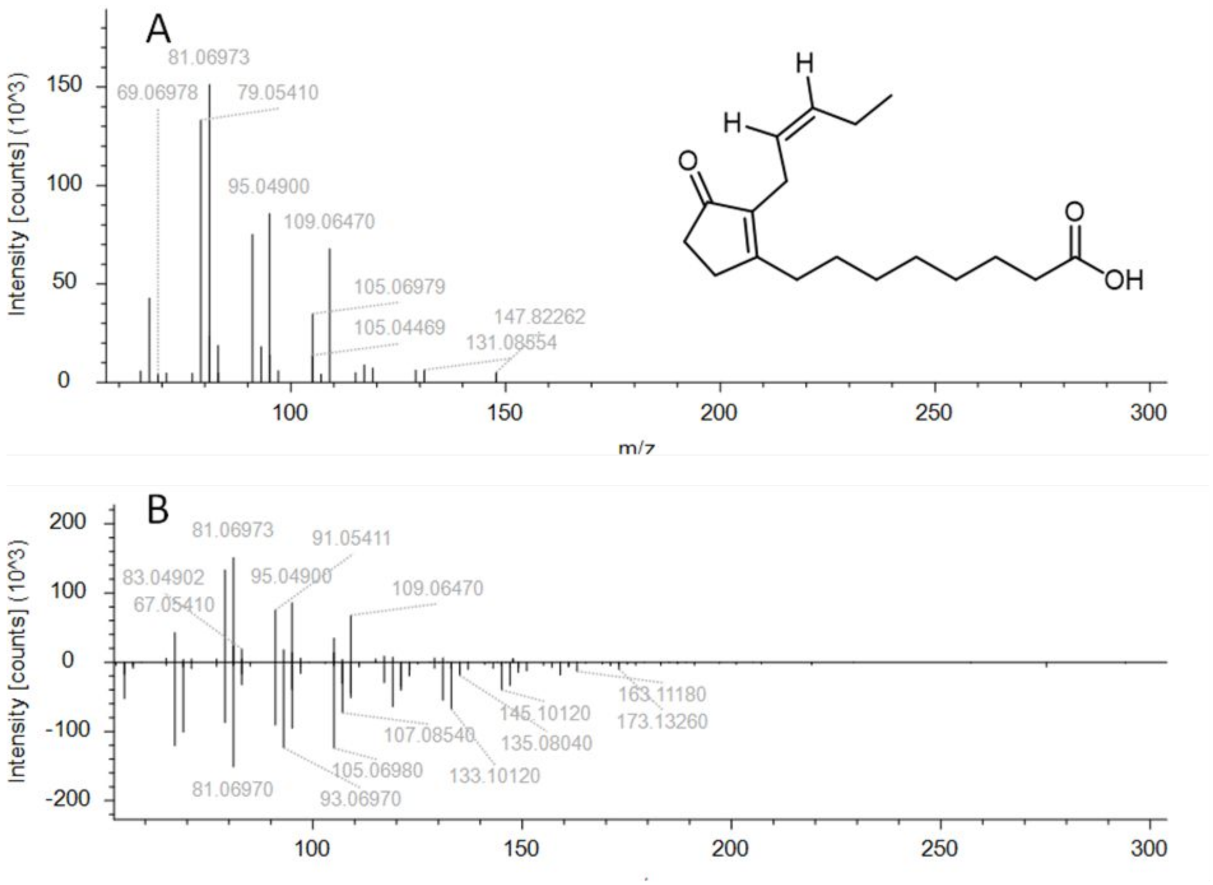

Fig. S7. (A) MS/MS spectrum of the ion with *m/z* 293.2111. **(B)** Spectral comparison between the obtained spectrum and the spectrum of cis-12-Oxo phytodienoic acid deposited in the MzVault.

9-KODE

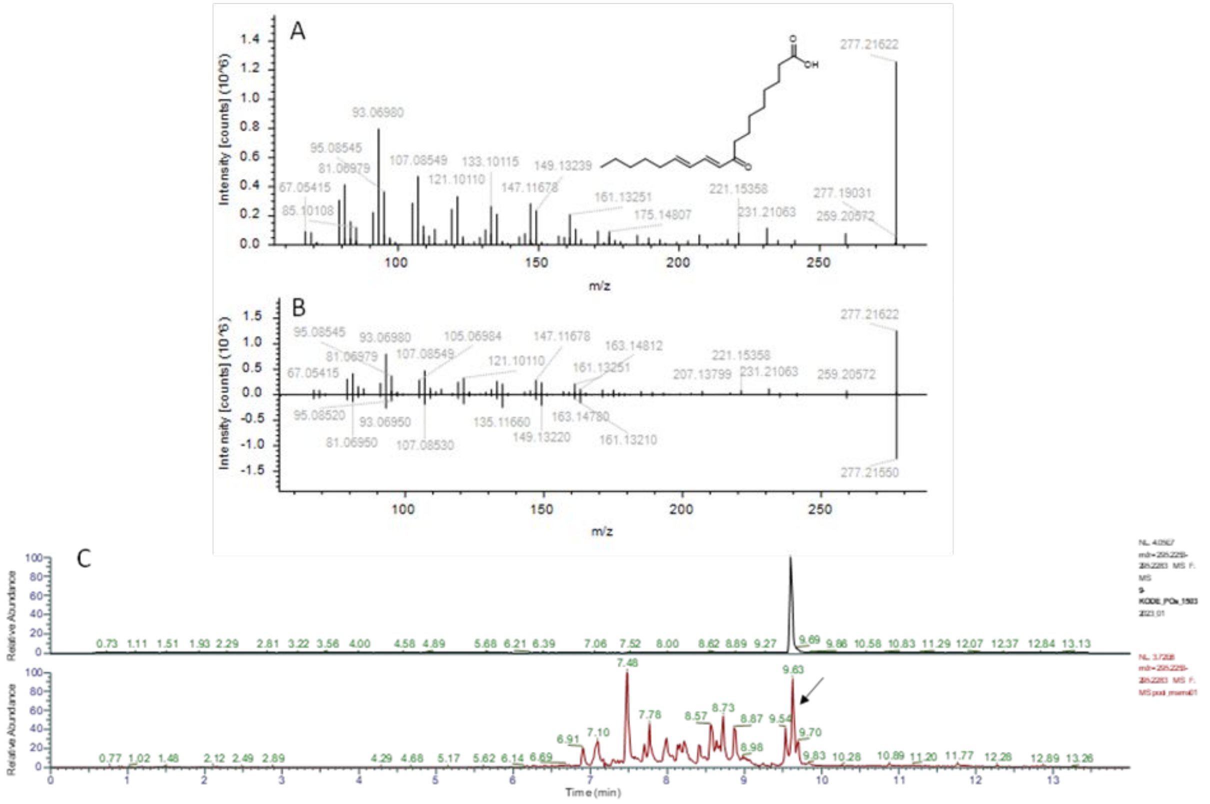

Fig. S8 (A) MS/MS spectrum of the ion with *m/z* 295.2268. **(B)** Spectral comparison between the obtained spectrum and the spectra of 9-KODE stored in the MzVault library. (C) Comparison of the retention time of the ion with *m/z* 295.2268 with the analytical standard of 9-KODE.

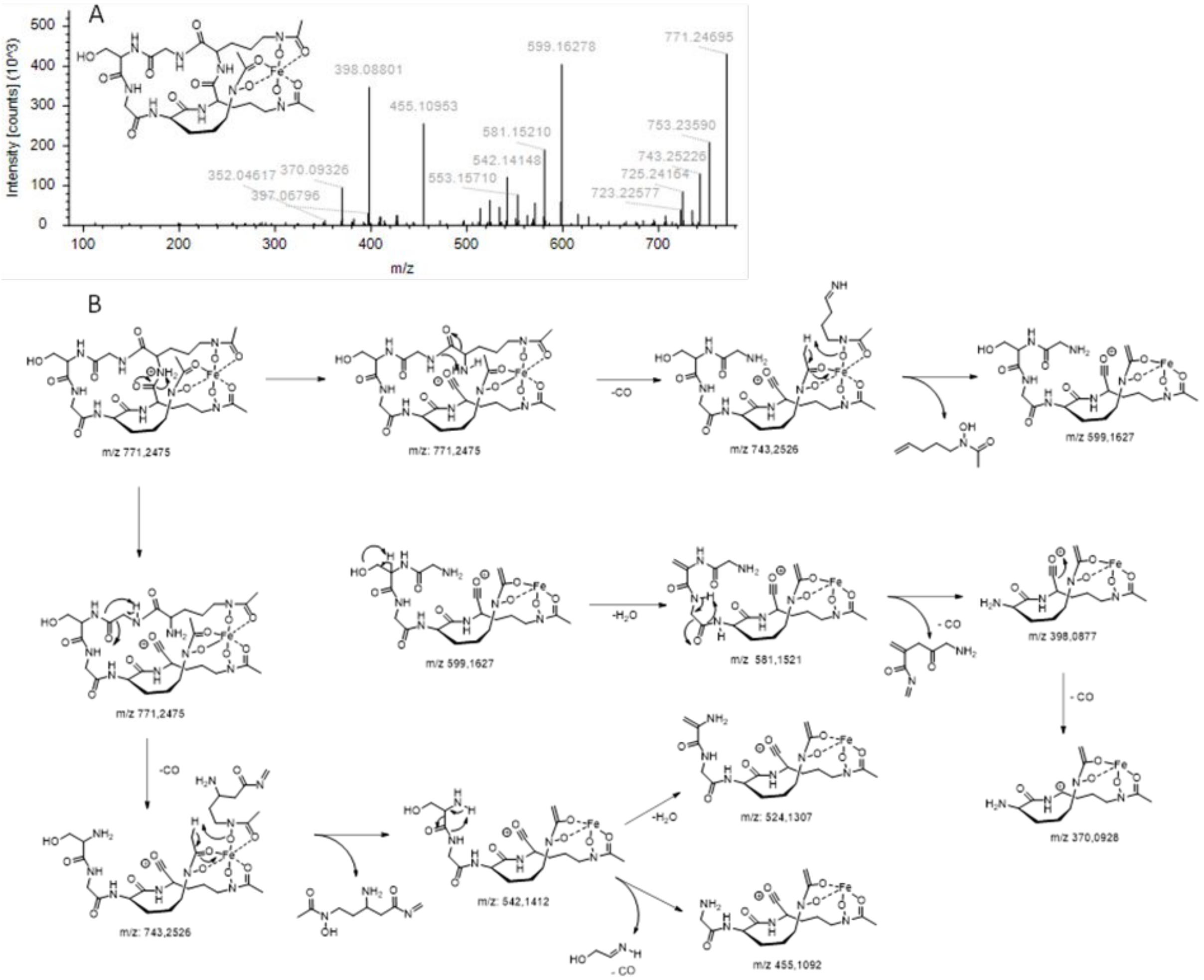

Fig S9. (A) MS/MS spectrum of the ion with *m/z* 771.2469. **(B)** Proposed fragmentation pathway for the most intense ions.

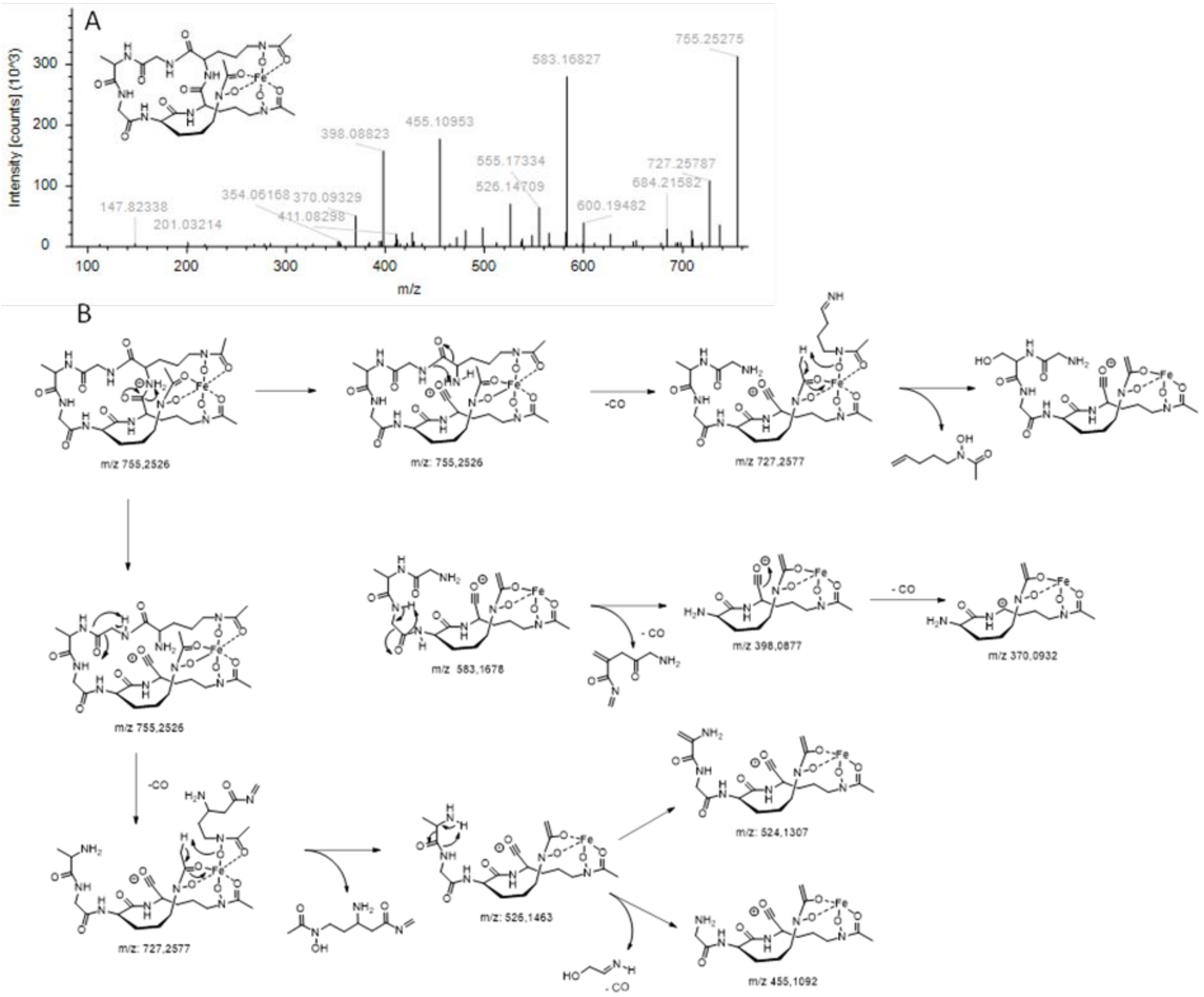

Fig. S10. (A) MS/MS spectrum of the ion with *m/z* 755.2527. **(B)** Proposed fragmentation pathway for the most intense ions.

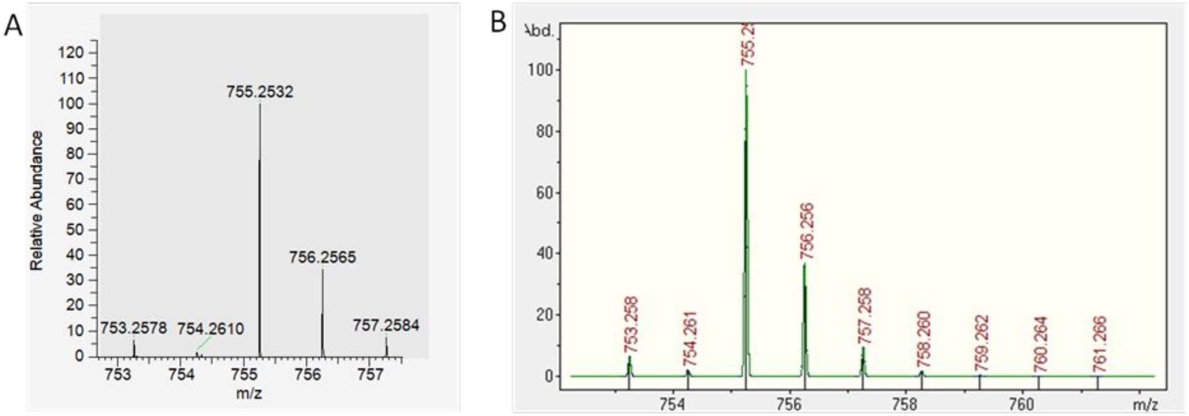

Fig. S11. (A) Full scan MS spectrum showing the isotopic pattern of the ion at *m/z* 755.2527 obtained. (B) Simulation of the isotopic pattern by the IsotopePattern Bruker software for the ion at m/z 755.2527.
